# Sensing Size through Clustering in Non-Equilibrium Membranes and the Control of Membrane-Bound Enzymatic Reactions

**DOI:** 10.1371/journal.pone.0143470

**Published:** 2015-12-14

**Authors:** Quentin Vagne, Matthew S. Turner, Pierre Sens

**Affiliations:** 1 Institut Curie, PSL Research University, CNRS, UMR 168, 26 rue d’Ulm, F-75005, Paris, France; 2 Dept. of Physics & Complexity Centre, University of Warwick, Coventry CV4 7AL, United Kingdom; Centrum Wiskunde & Informatica (CWI) & Netherlands Institute for Systems Biology, NETHERLANDS

## Abstract

The formation of dynamical clusters of proteins is ubiquitous in cellular membranes and is in part regulated by the recycling of membrane components. We show, using stochastic simulations and analytic modeling, that the out-of-equilibrium cluster size distribution of membrane components undergoing continuous recycling is strongly influenced by lateral confinement. This result has significant implications for the clustering of plasma membrane proteins whose mobility is hindered by cytoskeletal “corrals” and for protein clustering in cellular organelles of limited size that generically support material fluxes. We show how the confinement size can be sensed through its effect on the size distribution of clusters of membrane heterogeneities and propose that this could be regulated to control the efficiency of membrane-bound reactions. To illustrate this, we study a chain of enzymatic reactions sensitive to membrane protein clustering. The reaction efficiency is found to be a non-monotonic function of the system size, and can be optimal for sizes comparable to those of cellular organelles.

## Introduction

Cell membranes are highly heterogeneous and dynamic structures whose biological activity is regulated by lateral partitioning [1–3] and recycling [4, 5] of membrane components such as receptors and enzymes. Domains enriched in glycosphingolipids and cholesterol transiently assemble at the plasma membrane where they serve as signalling platforms by segregating membrane receptors [6]. These domains are thought to interact in a complex way with the cytoskeleton, which could play a role in regulating their size and dynamics, and therefore impact the biochemical activity of the plasma membrane [7–10]. Membrane heterogeneities have also been observed in cellular organelles such as the Golgi apparatus [11–13]. It has been argued that they play an important role in defining the biochemical identity of organelles and help regulate inter-organelle exchange [14]. As such, they could be involved in the mechanism by which the size of cellular organelles is sensed and controlled, an open problem in cell biology [15].

Spatial heterogeneities in cellular membranes are typically small and short-lived and differ from the relatively simple (equilibrium) thermodynamic behaviour of *in-vitro* artificial composite membranes. The latter generally exhibit a sharp transition between an homogeneous state and one that supports large and stable membrane macro-domains depending on the temperature and membrane composition [16–18]. The compositional diversity of cell membranes [19, 20] could be partly responsible for this difference but it is important to note that cell membranes are inherently out-of-equilibrium systems, experiencing continuous fluxes of matter and energy that may either drive or hinder spatial segregation. These fluxes might include the active transport of membrane proteins by the cytoskeleton [21], the recycling of membrane components by exocytosis and endocytosis [22] or the activation/inactivation cycle of GTPases such as Rab proteins [23].

Here, we study the effect of the recycling of membrane components on their ability to form membrane “clusters”, a generic term referring to either membrane heterogeneities, lipid domains or protein aggregates. Qualitatively, recycling reduces the lifetime of membrane components and therefore the likelihood that they meet preexisting clusters or nucleate new ones. In the steady-state the typical cluster size should therefore decrease with increasing recycling rate. Quantitative studies of this effect are often based on reaction-diffusion equations [24, 25], or the Smoluchowski coagulation equation with an external flux [26–29]. Such approaches neglect fluctuations due to the stochastic arrival and departure of components recycled in and out of the membrane, and are in principle valid when the system’s size goes to infinity. In this limit, a power-law dependence of the characteristic cluster size on the recycling rate is predicted, and for physiological recycling rates, the characteristic size if of the order of 1*μ*m [29]. This is comparable to the typical size of cellular organelles such as endosomes or the Golgi apparatus, indicating that finite size effects are expected to influence protein clustering in these organelles. Finite size effects should also affect the clustering of plasma membrane proteins corralled by cytoskeleton filaments, which display reduced mobility and are often confined to explore restricted membrane patches [7, 8]. In this paper we combine stochastic simulations with an analytical approach to properly account for the effect of lateral confinement on protein clustering. We show that finite-size effects qualitatively alter the size distribution of membrane heterogeneities such as protein clusters suggesting that the system size can be sensed (hence regulated) through the statistics of cluster formation. To illustrate the physiological relevance of our results, we show that the efficiency of enzymatic reactions that are sensitive to enzyme clustering strongly depends on the size of the compartment in which this reaction takes place. For reasonable parameter values, the efficiency is found to be optimal for a system size comparable to the size of cellular organelles.

## Model

We consider here a membrane system populated by a minority species *A* diffusing in a bulk of species *B* with a diffusion coefficient *D*. The system has a finite size, and contains a total number *N*_*s*_ of *A* and *B* molecules. This size is an important parameter of the model which corresponds to the area over which *A* components are free to diffuse. It can represent the area of the entire plasma membrane, the area of smaller cellular organelles, or a small region of the plasma membrane within which membrane components are confined, *e*.*g*. by the cytoskeleton.

We are studying an open system of finite size, where some of the components, those belonging to the minority phase, are subjected to fluxes coming in and out of the system. One must then specify how the size of the system is actually fixed, namely what is the response of the majority phase (B) to fluxes of the minority phase (A). The mechanisms by which cells regulate the size of their various organelles are complex and still poorly understood [15, 30, 31]. For the sake of generality we have studied two extreme scenarios of size regulation. In the “tight regulation” scenario, the system’s size (*N*_*s*_) remains constant at all time, which implies that fluxes of *B* exactly compensate fluxes of *A* at all time. In the “loose regulation” scenario, the amount of *B* components in the system remains constant at all time, so that the system’s size *N*_*s*_ fluctuates following the stochastic arrival and removal of *A* components. These two scenarios are discussed in detail in Section B in S1 File, and give identical results, provided that the *A* species is diluted enough. We thus concentrate here on the tight regulation scenario (*N*_*s*_ constant at all time).

The system dynamics is described as a sequence of discrete, stochastic events. The *A* molecules aggregate whenever they meet to form clusters of increasing sizes, which are recycled by vesicles budding off the membrane. We assume here for simplicity that spontaneous cluster fragmentation is unfavorable due to a high line tension so that growth by evaporation and condensation (Ostwald ripening) may be neglected [32]. In the following, areas are normalised by the area of a monomeric unit *s*, and time is expressed in unit of the diffusion time *τ*_*D*_ = *s*/*D*, where *D* is the diffusion coefficient of *A* components. For the sake of simplicity we do not keep track of the relative positions of the clusters and we simply estimate the diffusional rate of encounter. For 2D membranes and relatively small clusters [33], the coalescence rate between two clusters is independent of the cluster sizes and can be estimated by *D*/(*N*_*s*_ *s*) which reduces to 1/*N*_*s*_ using the chosen time unit.

**Fig 1:**
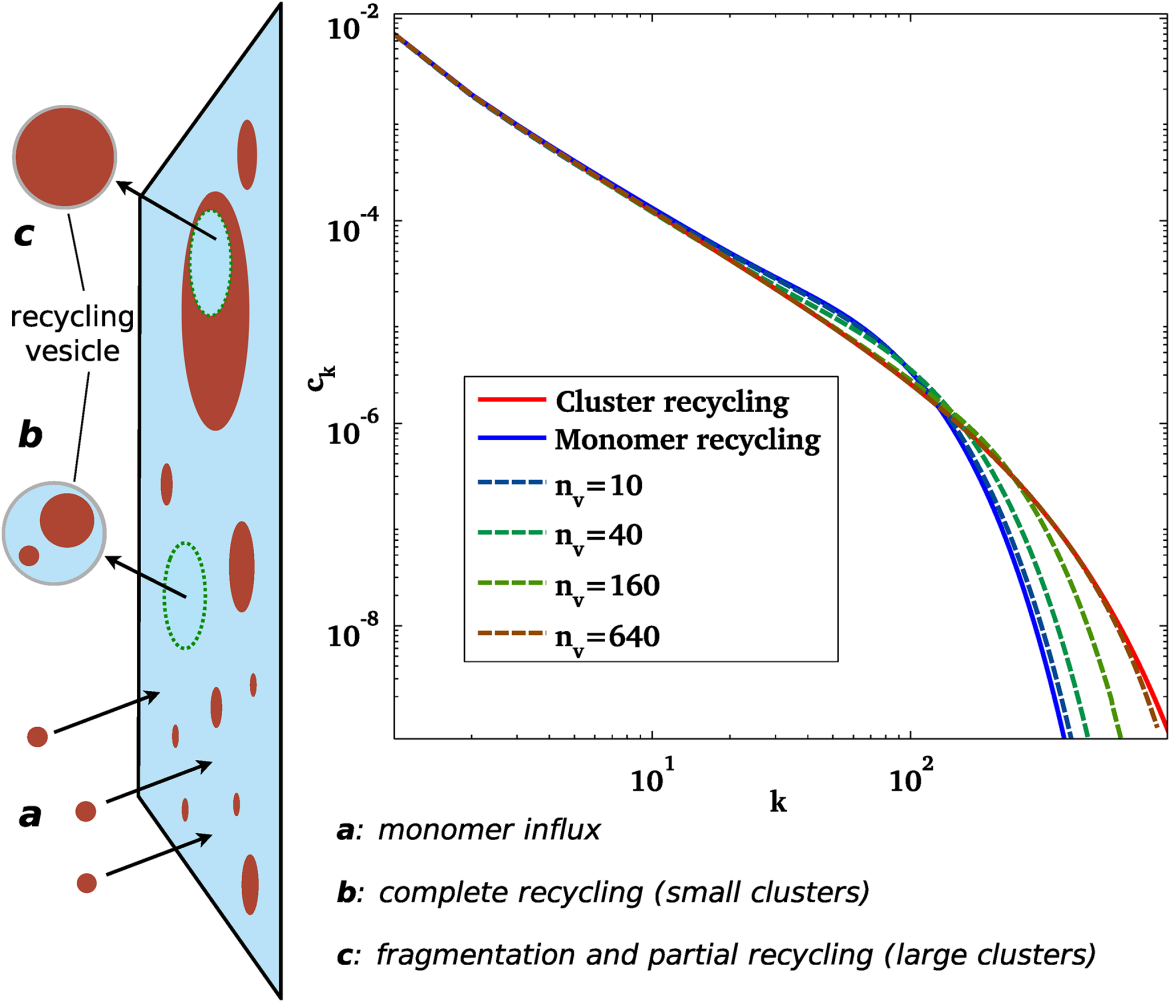
Left: Sketch of the recycling scheme: The minority phase (red) is brought to the membrane as monomers (a) and recycled by vesicles of a given size *n*_*v*_. Clusters of size smaller than *n*_*v*_ are recycled entirely in one vesicle (b), while larger clusters are fragmented and loose an area *n*_*v*_ during a recycling event (c). Right: Stationary cluster size distribution in the limit of infinite systems, for different values of the size of a recycling vesicle *n*_*v*_. These results are obtained by numerically solving the master equation (Eqs (2, 3)). The “whole cluster recycling” limit (*n*_*v*_ → ∞), corresponding to Eq 4, is shown in red. The “monomer recycling” limit (*n*_*v*_ = 1) is shown in blue. Intermediate values of *n*_*v*_ are the dashed lines.

The recycling dynamics is modelled as follows. We assume a constant influx *J* of monomer of the *A* species per unit area occupied by the *B* species, and the removal of *A*-clusters by recycling vesicles of a given size *n*_*v*_ (in monomer unit), budding off the membrane at a given rate *K*/*n*_*v*_ per unit area (see Fig 1). A small cluster of size *k* ≪ *n*_*v*_ is removed within a single recycling vesicles provided the vesicle buds anywhere within an area *n*_*v*_ around the cluster, giving a rate of small cluster removal *Kn*_*v*_/*n*_*v*_ = *K*. On the other hand, only an area *n*_*v*_ of larger clusters (with *k* ≫ *n*_*v*_) is removed in one vesicle recycling. This happens whenever a vesicle buds from the area *k* covered by the cluster, hence with a rate *Kk*/*n*_*v*_. Here, we use a phenomenological function *f*_*k*_ to reproduce the cross-over between these two regimes, and write the rate of cluster recycling *Kf*_*k*_ with the arbitrary choice $f_{k}={\left(1+{\left(k/n_{v}\right)}^{5}\right)}^{\frac{1}{5}}$. The complete system dynamics are defined by the following stochastic transitions:

$$\begin{array}{c} \left\{\begin{array}{cccc} \text{Injection}: & B & \overset{J}{\to } & A_{1}\\ \text{Growth}: & A_{k}+A_{l} & \overset{1/N_{s}}{\to } & A_{k+l}\\ \text{Removal}: & A_{k\leq n_{v}} & \overset{Kf_{k}}{\to } & kB\\ & A_{k>n_{v}} & \overset{Kf_{k}}{\to } & A_{k-n_{v}}+n_{v}B \end{array}\right. \end{array}$$ (1)

where *A*_*k*_ denotes a cluster with *k* monomers. Each arrow is to be understood as one possible stochastic event with the associated rate. The state of the system at time *t* is given by the set of stochastic variables Ω(*t*) = {*n*_1_(*t*), *n*_2_(*t*), …} (*n*_*k*_ being the number of clusters *A*_*k*_) that follows a Markovian evolution.

### Infinite membrane limit

Here we briefly discuss the limit of an infinite membrane, as it is under this assumption that a number of similar systems have been studied in the literature. We first define the dimensionless concentrations of clusters *c*_*k*_(*t*) = *n*_*k*_(*t*)/*N*_*s*_ for all sizes *k*. These concentrations are stochastic quantities fluctuating with time. However if the size *N*_*s*_ of the membrane goes to infinity the fluctuations become smaller and eventually disappear. The system is then fully described by the macroscopic concentrations *c*_*k*_(*t*) and loses its stochastic nature. Cluster growth may in this case be studied more simply using the general framework of the Smoluchowski coagulation equation [34]. The master equation for our system reads [29]:

$$\begin{array}{c} \frac{dc_{k}}{dt}=J_{k}-c_{k}\sum_{l=1}^{\infty }c_{l}+\frac{1}{2}\sum_{l=1}^{k-1}c_{l}c_{k-l} \end{array}$$ (2)

where *c*_*k*_ is the concentration of clusters containing *k* molecules of *A* and *J*_*k*_ represents the external fluxes, defined following Eq 1 as:

$$\begin{array}{c} J_{k}=J\left(1-\phi \right)\delta_{k,1}-K\left(f_{k}c_{k}-f_{k+n_{v}}c_{k+n_{v}}\right) \end{array}$$ (3)

where $\phi \equiv \sum_{1}^{\infty }kc_{k}$ is the area fraction of *A* components. At steady state, we can show that the surface fraction converges to *ϕ* = *J*/(*J* + *K*). The “vesicular recycling” scheme, defined by the crossover function *f*_*k*_, has two well defined limits; the “whole cluster” recycling scheme when all clusters are recycled with the same rate (*n*_*v*_ → ∞), and the “monomer recycling” scheme, where all monomers are recycled at the same rate, whether in clusters or not (*n*_*v*_ = 1). The whole cluster recycling scheme may be solved analytically (see Section A in S1 File) and is well approximated by a power-law with an exponential cut-off:

$$\begin{array}{c} c_{k}\approx \sqrt{\frac{\phi K}{2\pi }}k^{-\frac{3}{2}}\exp\left(-\frac{k}{\kappa }\right),\;\kappa =\frac{2\phi }{K} \end{array}$$ (4)

The cutoff size *κ* defines a natural cluster size that is completely determined by the recycling rates *J* and *K*[29]. The monomer recycling scheme is less amenable to analytical treatment, but shares the same features (see Fig 1), with an identical power law behaviour for small clusters and an exponential decay for large clusters. It is shown in Section A in S1 File that the cut-off size is in this case *κ*/2. Fig 1 shows the transition between monomer recycling and whole cluster recycling upon varying the typical recycling size *n*_*v*_ in Eq 3. The choice of function *f*_*k*_ is not expected to qualitatively affect the smooth transition observed in Fig 1.

One intuitively expects that this infinite membrane analysis might be applied to a finite system only when fluctuations are negligible, which ought not to be the case when the typical cluster size *κ* is of order or larger than the system size.

## Results

### Stochastic simulations of finite systems

We implement a full stochastic version of the non-equilibrium clustering process using a Gillespie algorithm [35] in order to study how the cluster size distribution is influenced by stochastic fluctuations and correlations occurring in systems of finite sizes. We concentrate on the “cluster recycling” and “monomer recycling” schemes previously described, for which the system dynamics satisfy simpler versions of Eq 1:

- for the whole cluster recycling scheme:
  $$\begin{array}{c} \left\{\begin{array}{cccc} \text{Injection}: & B & \overset{J}{\to } & A_{1}\\ \text{Growth}: & A_{k}+A_{l} & \overset{1/N_{s}}{\to } & A_{k+l}\\ \text{Removal}: & A_{k} & \overset{K}{\to } & kB \end{array}\right. \end{array}$$ (5)
- and for the monomer recycling scheme:
  $$\begin{array}{c} \left\{\begin{array}{cccc} \text{Injection}: & B & \overset{J}{\to } & A_{1}\\ \text{Growth}: & A_{k}+A_{l} & \overset{1/N_{s}}{\to } & A_{k+l}\\ \text{Removal}: & A_{k} & \overset{Kk}{\to } & A_{k-1}+B \end{array}\right. \end{array}$$ (6)

**Fig 2:**
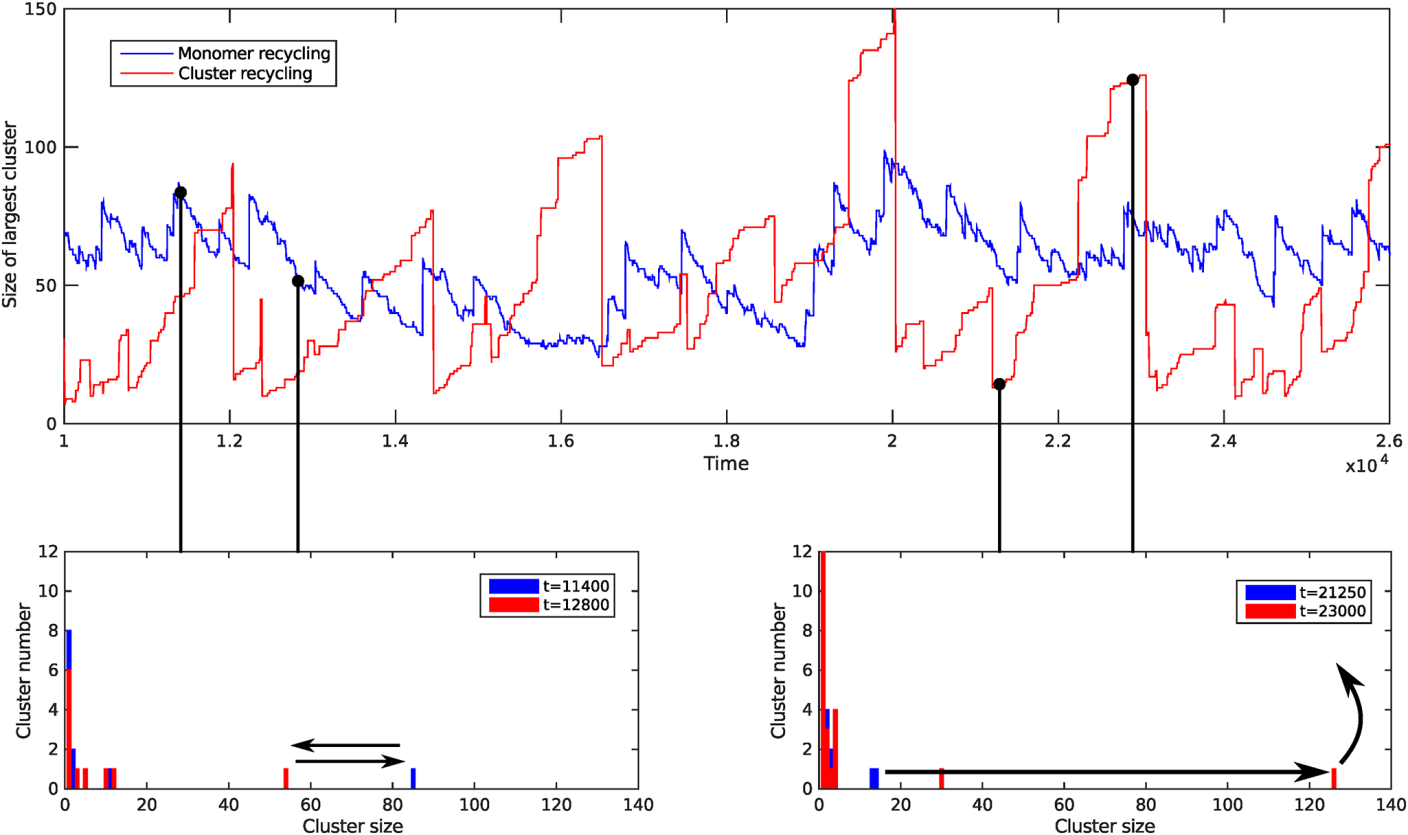
Bottom: Snapshot of the cluster size distribution at two different times for the monomer recycling scheme (left) and the whole cluster recycling scheme (right). The black arrows illustrate the dynamics of the large cluster. Top: Evolution of the size of the largest cluster with time for the two schemes. The parameters are *J* = 2.2 ⋅ 10^−4^ and *K* = 2 ⋅ 10^−3^ (whole cluster recycling) or *J* = 1.1 ⋅ 10^−4^ and *K* = 1 ⋅ 10^−3^ (monomer recycling). This ensure in both cases that *ϕ* = 0.1 and *κ* = 100. The system size is *N*_*s*_ = 1000.

**Fig 3:**
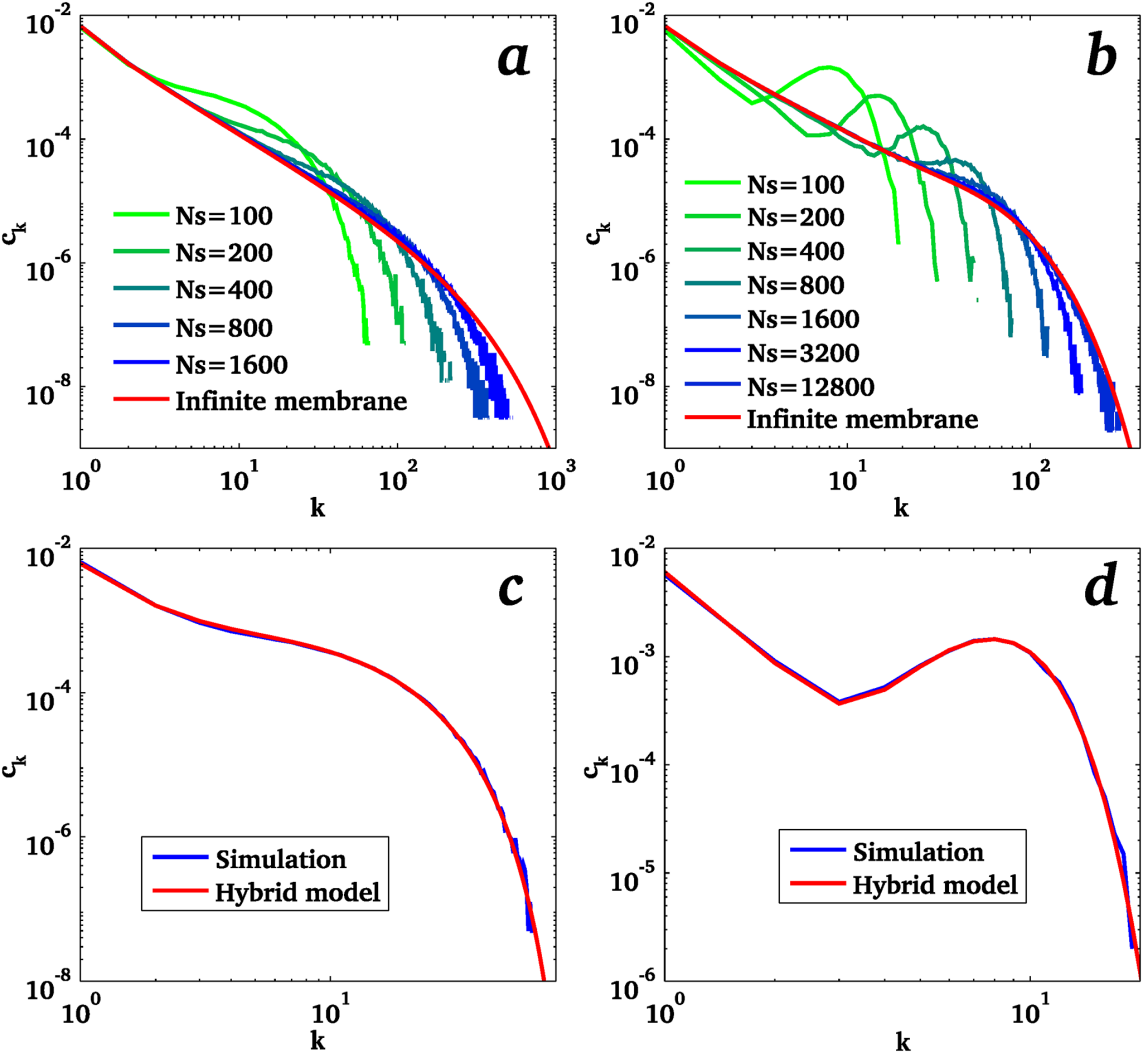
Steady-state distributions of cluster size for small systems, obtained by simulations (averaged over a large number of independent simulations) compared to the infinite membrane result. Simulations are shown for different system sizes for the whole cluster recycling (a) and monomer recycling (b) schemes. Comparison with the hybrid analytical model developed in the text for the whole cluster recycling (c) and monomer recycling (d) schemes show excellent agreement for strong confinement (with *N*_*s*_ = 100). All plots are for *ϕ* = 0.1 and *κ* = 180.

For system sizes large compared to the natural cluster size (*N*_*s*_ ≫ *κ*), the numerical results for the mean cluster size distribution at steady-state are in perfect agreement with the infinite membrane predictions from Eq 2 (see S1 Fig). However this is no longer the case when *N*_*s*_ is close to or smaller than the natural cluster size *κ*. Snapshots of the cluster size histograms (Fig 2) typically show one large cluster coexisting with a distribution of small clusters. Fig 2 also shows the evolution of the size of the largest cluster with time. The largest cluster grows continuously until being recycled in the whole cluster recycling scheme or shows smaller fluctuations around a stationary size dictated by a balance between growth and monomer recycling in the monomer recycling scheme. This observation supports the analytical treatments developed in the following sections. Direct comparison with the distribution from the infinite membrane can be obtained by averaging the instantaneous concentrations *c*_*k*_(*t*) = *n*_*k*_(*t*)/*N*_*s*_ (in time or over a large number of independent simulations). The steady-state average distributions are shown in Fig 3 for the two recycling schemes. The power-law predicted in the infinite membrane model (Eq 4) is still valid for small clusters of size *k* ≪ *ϕN*_*s*_, but large deviations are observed for larger clusters. Furthermore, the size distributions obtained in the monomer and whole cluster recycling schemes, which are qualitatively similar for large systems, show qualitative differences in finite systems. The former exhibits a broad shoulder for large clusters, while the latter exhibits a (broad) peak at a particular cluster size.

### Analytical model

The snapshots of stochastic simulations at a given time *t* (Fig 2) suggest an hybrid analytical model where the small clusters can be treated using the deterministic infinite membrane equation (Eq 2), while the large cluster requires a model that account for its stochastic growth and recycling. In the following, instead of introducing a specific size cut-off differentiating small and large clusters, we simply consider two coexisting populations. The first population is composed of many (small) clusters for which stochastic fluctuations are neglected. It can be described by average concentrations $c_{k}^{\prime }$ solutions of the continuous equation Eq 2. The second population is reduced to a single cluster whose size fluctuates stochastically. It is described by the probability *p*(*n*, *t*) to observe a cluster of size *n* at time *t*, hence an average concentration 〈*p*(*n*)〉/*N*_*s*_. In principle, the continuous distribution $c_{k}^{\prime }$ includes clusters whose size goes from 1 to infinity, and the size of the single stochastic cluster may also vary from 1 to infinity. In practice, the former distribution represents the small clusters, occupying an average surface fraction $\phi_{S}=\sum kc_{k}^{\prime }$, and the stochastic cluster is on average quite large compared to the continuous distribution. It occupies an average area fraction *ϕ*_*L*_. The full size distribution including both populations is then given by: $c_{n}=c_{n}^{\prime }+\langle p\left(n\right)\rangle /N_{s}$.

The size distribution of small clusters is obtained using the equation for an infinite membrane (Eq 2), modified to account for the fact that an average fraction *ϕ*_*L*_ of the membrane is occupied by a large cluster and for the fact that small clusters disappear both by direct recycling and by coalescence with the large cluster. Thus $c_{k}^{\prime }$ is simply given by Eq 4, in which *ϕ* is replaced by *ϕ*_*S*_ and *K* is replaced by *K* + 1/*N*_*s*_.

The stochastic evolution of the size *n*(*t*) of the large cluster is obtained as follows: We assume that the size distribution of small clusters adjust rapidly to the stationary distribution for any value of the surface fraction taken by the large cluster *ϕ*_*L*_ = *n*/*N*_*s*_. The rate at which a small cluster coalesces with the large one is 1/*N*_*s*_ and we assume for simplicity that each coalescence event with the large cluster increases its size by one monomer unit. Each recycling event either removes the entire cluster (whole cluster recycling), or reduces its size by one unit (monomer recycling):

$$\begin{array}{c} \left\{\begin{array}{cc} n\to n+1 & \text{At}\;\text{rate}\;\epsilon (N_{s}-n)\\ \text{and}\\ n\to 0 & \text{At}\;\text{rate}\;K\;(whole\;cluster\;recycling)\\ \text{or}\\ n\to n-1 & \text{At}\;\text{rate}\;nK\;(monomer\;recycling) \end{array}\right. \end{array}$$ (7)

with *ϵ* = *J*/(1 + *N*_*s*_(*J* + *K*)). The corresponding steady-state probability distributions are derived in Section B in S1 File, and are given by:

$$\begin{array}{c} p_{cluster}\left(n\right)\approx \frac{K}{\epsilon N_{s}}{\left(1-\frac{n}{N_{s}}\right)}^{\frac{K}{\epsilon }-1}\\ p_{monomer}\left(n\right)=\frac{\epsilon^{n}}{{\left(\frac{\epsilon }{K}+1\right)}^{N_{s}}K^{n}}\cdot \frac{N_{s}!}{n!\left(N_{s}-n\right)!} \end{array}$$ (8)

The average surface fraction occupied by each population is identical in both recycling schemes and given by:

$$\begin{array}{c} \phi_{S}=\phi \left(\frac{KN_{s}}{KN_{s}+1}\right),\;\phi_{L}=\phi -\phi_{S} \end{array}$$ (9)

Fig 3 shows a comparison between the hybrid model described above and the stochastic simulations. The agreement is excellent for both recycling schemes for small systems ($N_{s}/\kappa ≲1$). The common feature of both schemes is a strong reduction of large size clusters as compared to the infinite membrane results, and the concomitant increase of probability of intermediate size clusters. This probability smoothly decreases with the cluster size in the whole cluster recycling scheme, reflecting the fact that the large clusters continuously grow until they get abruptly recycled. In the monomer recycling scheme, a broad peak is visible at an intermediate cluster size, indicating smaller fluctuations of the size of the largest cluster. This difference is also apparent in the temporal evolution of the largest cluster size is shown Fig 2.

For larger systems, the size distribution predicted by the analytical treatment remains in excellent agreement with the numerics for the whole cluster recycling scheme, but deviations are observed for the monomer recycling scheme (see S3 Fig). In particular, the broad peak predicted by the analytical model disappears in the simulation. This discrepancy under moderate confinement is likely due to the failure of the approximation that there exists only a single large-size cluster.

It is noteworthy from Fig 3 that lateral confinement of membrane proteins (finite size effects) affect the average probability of all but the smallest protein clusters present on the membrane. One may thus expect confinement to impact on any physiological activity of the membrane that relies on clustering of membrane components. In order to illustrate the biological relevance of this results, we study how the system’s size impact the efficiency of a model of enzymatic reactions taking place inside clusters.

### Application to enzymatic reactions

The co-localisation of enzymes in membrane compartments is thought to improve the efficiency of membrane-bound enzymatic reactions, in particular those involving several steps. If the product of a reaction is the substrate of a subsequent reaction, enzyme clustering can prevent the loss of the intermediate product by diffusion and degradation (a concept sometimes termed metabolic channeling) [36, 37]. Moreover, it was recently shown that maximum reaction efficiency and reliability can occur at an optimal cluster size [38, 39]. The results presented above and summarised in Fig 3 show that the size distribution of membrane clusters is strongly affected by lateral confinement. This raises the exciting possibility that the size of the system could influence, and thus be sensed through, the efficiency of biochemical reactions requiring enzyme clustering. In the cellular context, the system size can represent either the size of an organelle in the membrane of which a reaction takes place, or the size of corrals hindering protein diffusion at the plasma membrane.

**Fig 4:**
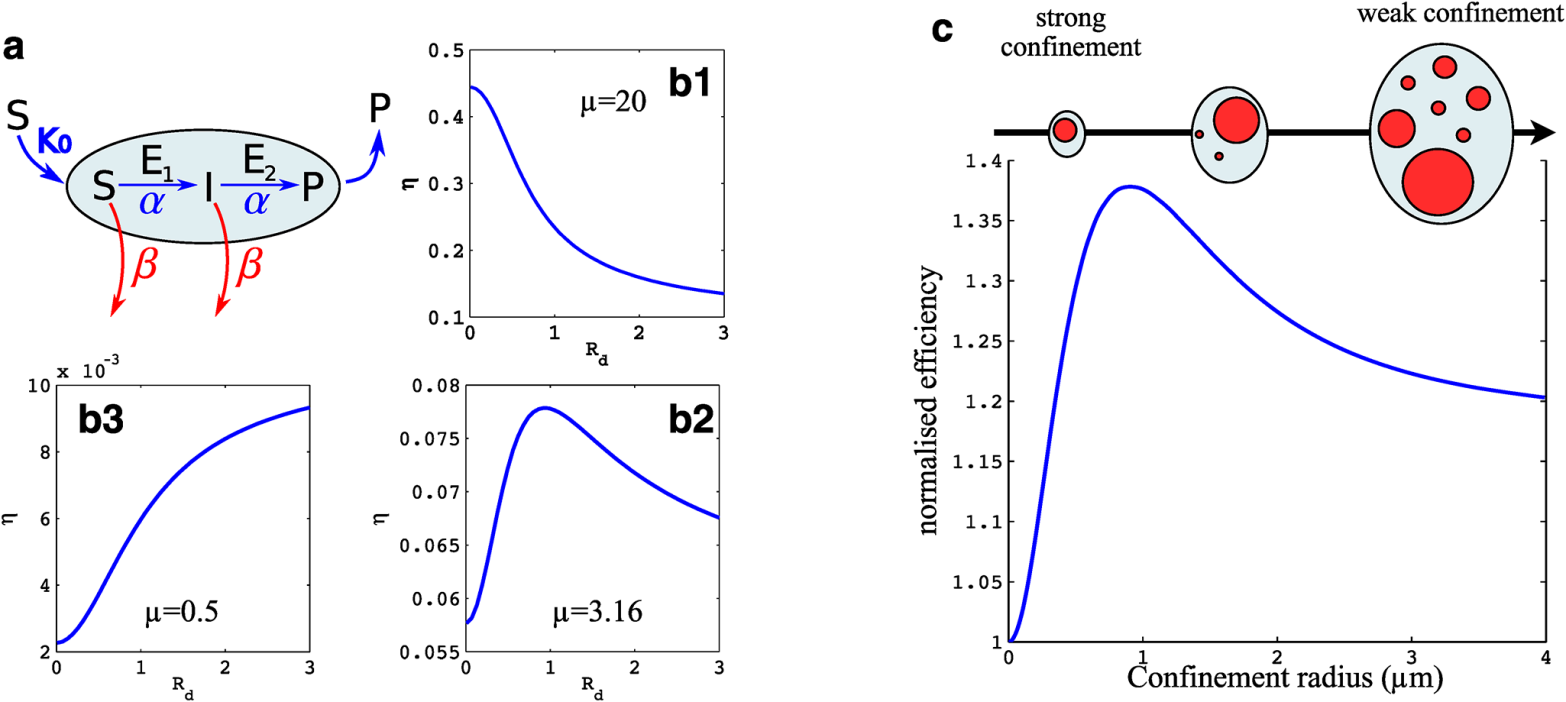
Efficiency of a two-step enzymatic reaction taking place on the membrane of an organelle as a function of the organelle’s size. (a): Sketch of the reaction described in the text, in which a substrate *S* is transformed into a product *P* via and intermediate *I*, each reactions being catalysed by different enzymes *E*_1_ and *E*_2_, both confined within clusters. (b1–b3) Reaction efficiency for a single membrane unit, defined as a circular cluster of size *R*_*d*_ in a circular membrane patch of size $R_{c}=R_{d}/\sqrt{\phi }$, for *ϕ* = 0.1 and for different value of the ratio of reaction to degradation rates *μ* (*R*_*d*_ is given in unit of $\sqrt{D_{m}/\beta }$). (c) Average reaction efficiency for the full systems, consisting of clusters with fluctuating sizes. The average size distribution (sketched) depends on the level of confinement (the system’s size), as quantified in Fig 3. The average efficiency η* (Eq 13) is normalised by that of an homogeneous system *η*_0_ Eq 10. All parameters are given in the text.

To explore this, we consider a generic two-step reaction similar to the one studied in [37–39] and shown in Fig 4a: a substrate *S* is transformed by enzyme *E*_1_ into an intermediate *I* that is then transformed by enzyme *E*_2_ into the product *P*. Metabolic channeling is obtained by assuming that the enzymes co-localize within clusters. For simplicity, we assume that there are no enzymes outside the clusters and that their concentration within clusters is fixed to a given value. They are also considered to process the substrate and intermediate at the same rate *α*. The substrate is brought to the membrane homogeneously with a flux *K*_0_ per unit area, and both substrate *S* and intermediate *I* are removed from the membrane at the same rate *β*. Removal may be due to stochastic detachment from the membrane, reaction with another competing pathway or thermally activated spontaneous change of conformation. In this framework, the total number of enzymes in the system is proportional to the area occupied by all clusters. Therefore the average number of enzymes per unit of membrane area is proportional to the cluster surface fraction *ϕ*.

The reaction efficiency *η* is defined as the ratio of the number of product molecules synthesised per unit area and time over the incoming flux of substrate molecules per unit area. The efficiency can be computed analytically in the absence of clustering (denoted *η*_0_), *i*.*e* for enzymes homogeneously distributed over the whole membrane, and for the case of complete clustering, where all enzymes are inside a single large cluster (denoted *η*_∞_). In an homogeneous system (no clustering), a successful reaction is the result of two (identical) reactions where the rate at which an enzyme is found and reacted with (*ϕα*) competes with the rate of degradation (*β*). If all enzymes are clustered into a single large cluster (perfect clustering), a successful reaction requires first that the substrate is delivered into the cluster (this has a probability *ϕ*). It is then the result of two (identical) reactions where catalysis (rate *α*) competes with degradation (rate *β*). The full derivation, given in the Section C in S1 File, leads to:

$$\begin{array}{c} \eta_{0}={\left(\frac{\phi \mu }{1+\phi \mu }\right)}^{2}\;,\;\eta_{\infty }=\phi {\left(\frac{\mu }{1+\mu }\right)}^{2} \end{array}$$ (10)

where *μ* ≡ *α*/*β* is the ratio of the reaction rate to the degradation rate.

Enzyme co-localisation into clusters is beneficial because it increases the likelihood that the intermediate *I*, produced inside a cluster by an enzyme *E*_1_, meets an enzyme *E*_2_ before it gets degraded. On the other hand, clustering leaves large membrane patches free of enzymes, which increases the likelihood that the substrate *S* is degraded before it meets an enzyme *E*_1_. One intuitively expects that the balance between these competing effects favours clustering if *μ* and *ϕ* are very small, since most substrate and intermediate molecules are degraded before encountering an enzyme in this case. Analysis of Eq 10 indeed shows that *η*_∞_ > *η*_0_ if *ϕμ* ^2^ < 1.

The efficiency of enzymatic reaction for the full range of cluster sizes may be derived be dividing the system into subunits of size *R*_*c*_ containing one cluster of size $R_{d}≃R_{c}\sqrt{\phi }$ surrounded by a membrane free of enzymes. The concentrations *c*_*S*_(*r*) and *c*_*I*_(*r*) of *S* and *I* are given by classical reaction-diffusion equations:

$$\begin{array}{ccc} \frac{dc_{S}}{dt} & = & K_{0}-\beta c_{S}-c_{S}\alpha \Theta \left(R_{d}-r\right)+D_{m}\Delta c_{S}\\ \frac{dc_{I}}{dt} & = & -\beta c_{I}+\left(c_{S}-c_{I}\right)\alpha \Theta \left(R_{d}-r\right)+D_{m}\Delta c_{I} \end{array}$$ (11)

where *D*_*m*_ is the diffusion coefficient of *S* and *I* and Θ is the Heaviside step function, expressing the fact that enzymes are fully segregated inside clusters. The efficiency of the subunit is:

$$\begin{array}{c} \eta \left(R_{d},\phi \right)=\frac{\alpha \int_{r<R_{d}}d\vec{r}c_{I}\left(\vec{r}\right)}{K_{0}\pi {R_{c}}^{2}} \end{array}$$ (12)

The efficiency of a single cluster depends on two parameters: *ϕ* and *μ*, in addition to the dimensionless cluster size ${\bar{R}}_{d}\equiv R_{d}\sqrt{\beta /D_{m}}$. It is derived in Section C in S1 File, and shown on Fig 4b for different value of the ratio of reaction to degradation rates *μ*. The asymptotic efficiencies given by Eq 10 for vanishingly small clusters (corresponding to an homogeneous membrane) and one large cluster are recovered in the limit of ${\bar{R}}_{d}\ll 1$ and ${\bar{R}}_{d}\gg 1$. As expected from the analysis of Eq 10, we find that full segregation is more efficient than no segregation at all if *μϕ* ^2^ < 1. Remarkably, we find that optimal efficiency is obtained for a finite cluster size if the parameters satisfy both *μ* > 5/4 and *ϕμ* < 1 (this is demonstrated in Section C in S1 File). This situation, corresponding to a relatively small number of enzymes performing at high reaction rate, is likely to be of physiological interest.

The results of the previous section show that when membrane clusters undergo coalescence and recycling, the cluster size distribution *c*_*k*_ is influenced both by the recycling kinetics and by the system’s size, as shown in Fig 3. In order to derive the efficiency of the two-step enzymatic reaction in this case, we assume that clusters diffuse and are recycled slowly compared to the kinetics of enzymatic reaction, so that the cluster size distribution is stationary at the time scale of enzymatic reaction. In this case, the efficiency for the full distribution of cluster size can be approximated by:

$$\begin{array}{c} \eta^{*}\left(N_{s},\phi \right)=\frac{1}{\phi }\sum_{k=1}^{N_{s}}kc_{k}\eta \left(k,\phi \right)\;,\;\phi =\sum_{k=1}^{N_{s}}kc_{k} \end{array}$$ (13)

where *η*(*k*, *ϕ*) is given by Eq 12 with $k=\pi R_{d}^{2}/s$. As discussed in Section C in S1 File, the efficiency given Eq 13 should be seen as the time average of the instantaneous efficiency, which follows the fluctuation of the cluster size distribution.

The variation of the efficiency with the system size must be evaluated numerically. It is shown on Fig 4c for a particular set of parameters. For the membrane parameters, we choose a unit size *s* = 1nm^2^[40] and a cluster diffusion coefficient *D* = 0.1 μm^2^ s^−1^[33], giving a typical diffusion time *τ*_*D*_ = *s*/*D* ≃ 10^−5^ s. Cellular organelles (the Golgi apparatus or endosomes) typically have their membrane fully renewed every few minutes [41]: *K* = 0.1 min^−1^. Choosing *J* = 10^2^ μm^−2^ s^−1^ gives a surface fraction *ϕ* ≃ 5% and a cluster cutoff size (for an infinite system) $\sqrt{s\kappa /\pi }≃1.5\;\mu m$. For the enzymatic reaction, we choose a diffusion coefficient *D*_*m*_ = 1 μm^2^ s^−1^, ten times larger than the cluster diffusion coefficient *D* in order to be in the range of validity of our approximation, but within a biologically relevant regime. We further choose *β* = 5 s^−1^ and *α* = 25 s^−1^ which gives *ϕ* < 1/*μ* = 0.2. With this choice of parameters, the efficiency of a single unit (Eq 12) shows a maximum for intermediate-size clusters *R*_*d*_ = 0.28 μm. The average efficiency over the entire membrane decorated with clusters satisfying the steady-state size distribution computed earlier and shown in Fig 3a (whole cluster recycling scheme) is shown in Fig 4c as a function of the system size. Since the system size tunes the cluster size distribution, the average efficiency also shows a maximum for intermediate system size (of order 0.9 μm for these parameters). This size scale is physiologically relevant and is close to the typical size of cellular organelles such as the Golgi apparatus. In the example presented, the improvement is ≃40% compared to an homogeneous distribution of enzymes and ≃20% compared to an infinite system. We note that the efficiency gain at the maximum can be made arbitrarily large by reducing the concentration of enzymes at the membrane.

## Discussion

The key result shown in Fig 4 is that enzymatic production, which is a local process, is sensitive to the size of the compartment in which it takes place. This behaviour stems from two very generic phenomena, namely the sensitivity of a two (or more) step enzymatic reactions to enzyme clustering, and the system size dependence of the cluster size distribution in non-equilibrium membranes. This result represents a new mechanism by which the size of organelles could be sensed and regulated, which remains a very basic open question in cell biology [15]. This mechanism is particularly useful in discriminating between one large organelle and several smaller ones: For the same total area, the rate of production of signalling molecules would be identical in both cases if it were solely dependent on the enzyme concentration. Within the mechanism we study the rate of production *per enzyme* is controlled by the compartment size, and can thus be used as a size sensor.

The phenomenon described here is quite general and does not rely on the details of either the recycling mechanism or the enzymatic chain, provided it involves several reactions. Our model can be extended to other biological processes thought to be controlled by the interplay between clustering and recycling, including the E-cadherin supramolecular organisation at cell-cell junctions [42] and the control of cell adhesion and motility by integrin clustering and endocytosis [43]. Experimental test of this model could involve in-vivo monitoring the spatial organization of cell surface and its enzymatic activity under controlled confinement. Such controlled confinement may be obtained with the combination of solid-state nanolithography to create obstacles on a substrate and supported lipid membrane techniques [44]. Finally, the formalism presented here is designed for biological membranes undergoing recycling, but our conclusions are more general and could be applied to other systems presenting non-equilibrium steady-states. For instance, the optimisation of the reactor size for maximum efficiency illustrated by Fig 4 could be relevant to the growing area of lab-on-chip microfluidic devices where chemical reactions take place in micro droplets of controllable sizes.

## Supporting Information

S1 File Analytical results and simulations. Analytical computations of steady-state size distributions are derived. Comparisons between numerical simulations and analytical results are shown. A detailed computation of the reaction efficiency *η* is presented.
(PDF)

S1 Fig Steady-state cluster size distribution: comparison between stochastic simulations and analytical results for infinite membranes. Domain size distributions at steady-state (*t* = 5.10^4^, averaged over 20000 realisations) for the fixed and fluctuating area models with *ϕ* = 0.1 and *κ* = 200. The membrane size is either fixed at *N*_*s*_ = 4000 or initially set at *N*_*s*0_ = 4000. The red line is the infinite membrane result.
(PDF)

S2 Fig Steady-state cluster size distribution for finite-size membranes. Steady-state cluster size distributions obtained by simulations compared to the infinite membrane result (in green). (A) Results for the fixed area model (*N*_*s*_ = 600, *κ* = 2000), for the steady-state surface fraction *ϕ* equal to 0.1 (blue), 0.5 (black) and 0.9 (red). Panels A1–A3: Comparison of the simulation results (blue) with the analytical predictions of Eqs (23, 24) of S1 File. (red) for the three surface fractions. (B) Results for the fluctuating area model (*ϕ* = 0.1, *κ* = 2000) with the initial membrane size *N*_*s*0_ equal to 300 (black), 600 (blue) and 1200 (red). Results (not shown) at other surface fractions show no qualitative difference. Panels B1–B3: Comparison of the simulation results (blue) with the analytical predictions of Eqs (29, 32) of S1 File (red) for the three initial sizes.
(PDF)

S3 Fig Steady-state cluster size distribution for finite-size membranes: accuracy of the hydric model. Steady-state size distributions using the monomer recycling mechanism for a fixed area. The injection rate is *J* = 10^−4^ and the recycling rate is *K* = 10^−3^. On the left panel the system size is set to *N*_*s*_ = 200 and on the right panel the system size is set to *N*_*s*_ = 800. The blue curves are stochastic simulation results extracted from Fig 2b of the main text and the red curves are the results of the analytical study.
(PDF)
