# Supplementary material for: Sensing Size through Clustering in Non-Equilibrium Membranes and the Control of Membrane-Bound Enzymatic Reactions
Source: PLoS One. 2015 Dec 14;10(12):e0143470. doi: 10.1371/journal.pone.0143470 (PMC4687633; doi:10.1371/journal.pone.0143470)
Supplement: S1 File — Analytical computations of steady-state size distributions are derived. Comparisons between numerical simulations and analytical results are shown. A detailed computation of the reaction efficiency η is presented. (PDF) [file pone.0143470.s001.pdf]

# Supplementary Information for : 'Sensing size through clustering in non-equilibrium membranes and the control of membrane-bound enzymatic reactions.'

Quentin Vagne, Matthew S. Turner and Pierre Sens

November 19, 2015

## Contents

**A Infinite membrane results** **2**

A.1 Master equation in the case of a fluctuating area . . . . . . . . . . . . . . . . . . . . . . 2

A.2 Steady-state size distribution: "whole cluster" recycling . . . . . . . . . . . . . . . . . 3

A.3 Steady-state size distribution: "monomer cluster" recycling . . . . . . . . . . . . . . . 4

A.4 Steady-state size distribution: vesicular recycling . . . . . . . . . . . . . . . . . . . . . 5

A.5 Stochastic simulations : Validation of the infinite membrane prediction for large membrane sizes . . . . . . . . . . . . . . . . . . . . . . . . . . . . . . . . . . . . . . . . . 5

**B Stochastic model for finite size systems** **5**

B.1 Whole cluster recycling . . . . . . . . . . . . . . . . . . . . . . . . . . . . . . . . . . . 6

B.1.1 Analytical model for finite size effects - fixed area . . . . . . . . . . . . . . . . 6

B.1.2 Analytical model for finite size effects - fluctuating area . . . . . . . . . . . . 7

B.1.3 Comparison with the numerical solution . . . . . . . . . . . . . . . . . . . . . 8

B.2 Analytical model for the monomer recycling scheme . . . . . . . . . . . . . . . . . . . 8

**C Computation of reaction efficiency** **10**

C.1 Inside a subunit of the membrane . . . . . . . . . . . . . . . . . . . . . . . . . . . . . . 10

C.2 Efficiency definition and computation in the homogeneous case . . . . . . . . . . . . 11

C.3 Expression of $\eta$ . . . . . . . . . . . . . . . . . . . . . . . . . . . . . . . . . . . . . 11

C.4 Qualitative and quantitative features of $\eta(R_d)$ . . . . . . . . . . . . . . . . . . . . . . 12

C.5 Computation of the global efficiency $\eta^\star$ . . . . . . . . . . . . . . . . . . . . . . . . . 13

# A Infinite membrane results

In this section, we first show that the infinite membrane formulation of the Smoluchowski coagulation equation is identical for both the fixed area model and the fluctuating area model. Then we give a detailed account of the stationary cluster size distribution for an infinite system under the two extreme scale-free recycling schemes, namely the "whole cluster recycling" and the "monomer recycling" schemes. We also show that the more complex "vesicular recycling" scheme, in which clusters are recycled by vesicles of a given size and larger clusters may be fragmented by recycling, allows for a smooth crossover between these two regimes upon varying the vesicle size from a monomeric size to infinity.

## A.1 Master equation in the case of a fluctuating area

Here we derive the master equation in the case of a fluctuating area, using the specific example of whole cluster recycling. In the case of a fluctuating area, A components are added to or removed from the membrane without any compensatory flux of B components. The area occupied by the $B$ component is constant and called $N_{s0}$. Monomers are injected at a constant rate and can get recycled or can coalesce with one another according to the laws given below :

$$\begin{cases} \text{Injection :} & \begin{pmatrix} n_1 \\ N_s \end{pmatrix} \xrightarrow{JN_{s0}} \begin{pmatrix} n_1+1 \\ N_s+1 \end{pmatrix} \\ \text{Growth :} & \begin{pmatrix} n_k \\ n_l \\ n_{k+l} \\ N_s \end{pmatrix} \xrightarrow{f(n_k,n_l,N_s)} \begin{pmatrix} n_k-1 \\ n_l-1 \\ n_{k+l}+1 \\ N_s \end{pmatrix} \\ \text{Removal :} & \begin{pmatrix} n_k \\ N_s \end{pmatrix} \xrightarrow{Kn_k} \begin{pmatrix} n_k-1 \\ N_s-k \end{pmatrix} \end{cases} \tag{S1}$$

Where the coalescence rate $f(n_k, n_l, N_s)$ is determined as follows. The typical dimensionless time to explore the area of the whole system is $N_s$, hence any given couple of cluster will coalesce at an average rate $1/N_s$. Thus, the rate of coalescence between clusters of size $k$ and clusters of size $l$ is given by :

$$f(n_k, n_l, N_s) = \frac{n_k n_l}{N_s}(1-\delta_{k,l}) + \frac{n_k(n_k-1)}{2N_s}\delta_{k,l} \tag{S2}$$

We want to derive an equation for $c_k(t) = \left\langle \frac{n_k(t)}{N_s(t)} \right\rangle$. In the infinite membrane approximation we neglect correlations and fluctuations, therefore we assume that $c_k = \frac{\langle n_k(t)\rangle}{\langle N_s(t)\rangle}$ and that $\langle n_k(t) n_l(t)\rangle = N_s(t)^2 c_k(t) c_l(t)$. Moreover, the average surface fraction $\phi(t) = \sum_k k c_k$ is related to the average membrane size by $\phi = 1 - \frac{N_{s0}}{\langle N_s(t)\rangle}$. Under these assumptions we can write the coalescence rates using products of concentrations, and the time derivative of $c_k(t)$ is :

$$\dot{c}_k(t) = \frac{1}{N_{s0}(1-\phi(t))} \cdot \frac{d\langle n_k\rangle}{dt} - c_k(t)\frac{\dot{\phi}(t)}{1-\phi(t)} \tag{S3}$$

Using the rates defined in Eq.(S1) to compute $\frac{d\langle n_k\rangle}{dt}$, Eq.(S3) leads directly to:

$$\begin{aligned} \dot{c}_k(t) &= J(1-\phi(t))\delta_{k,1} - \frac{\dot{\phi}(t)}{1-\phi(t)} c_k \\ &\quad - K c_k - c_k \sum_{l=1}^{\infty} c_l + \frac{1}{2}\sum_{l=1}^{k-1} c_l c_{k-l} \end{aligned} \tag{S4}$$

At steady state, the term in $\dot{\phi}$ disappears from the equation, and the fluctuating area model is characterised by the same (steady-state) equation than the fixed area model (Eqs.(1,2) in the main text, written here for the "whole cluster recycling" limit: $n_v \to \infty$).

## A.2 Steady-state size distribution: "whole cluster" recycling

Here we compute exactly the cluster size distribution $c_k$ at steady-state in the infinite membrane approximation. We therefore look for the solution of :

$$0 = J(1-\phi)\delta_{k,1} - K c_k - c_k \sum_{l=1}^{\infty} c_l + \frac{1}{2}\sum_{l=1}^{k-1} c_l c_{k-l} \tag{S5}$$

Here, $J$ is the monomer injection rate per site and $K$ is the cluster recycling rate. As in the main text, time is rescaled by the characteristic coalescence time, limited by diffusion: $\tau_D = s/D$, where $s$ is the area of one monomer. The total surface fraction $\phi = \sum_1^{\infty} k c_k$ can be obtained by summing Eq.(S5) multiplied by $k$ over all $k$. The total concentration of clusters of any size $C = \sum_1^{\infty} c_k$ is obtained by summing Eq.(S5) alone over all $k$ and using the expression of $\phi$:

$$\phi = \frac{J}{J+K} \tag{S6}$$

$$C = \sqrt{K^2 + 2\phi K} - K \tag{S7}$$

Now, we can obtain $c_k$ recursively as a function of $C$ using Eq.(S5) :

$$c_k = U_k \frac{(\phi K)^k}{(K+C)^{2k-1}} \tag{S8}$$

In which $U_k$ is a numerical factor given by $U_k = \frac{(2k-2)!}{2^{k-1}k!(k-1)!}$. Replacing $C$ by its value obtained in Eq.(S7) gives the explicit solution :

$$c_k = \sqrt{\phi K}\frac{U_k}{\sqrt{\frac{K}{\phi}+2}^{\,2k-1}} \tag{S9}$$

For $k \gg 1$ we have $U_k \approx \frac{2^k}{2\sqrt{\pi}} k^{-\frac{3}{2}}$. In the limit $\frac{K}{\phi} \ll 1$ and $k \gg 1$ one can get the following approximation for $c_k$ :

$$c_k \approx \sqrt{\frac{\phi K}{2\pi}} k^{-\frac{3}{2}} \exp\left(-\frac{k}{\kappa}\right), \quad \kappa = \frac{2\phi}{K} \tag{S10}$$

### A.3 Steady-state size distribution: "monomer cluster" recycling

We suppose that each monomer inside a cluster of size $n$ can be recycled at a rate $K$. Therefore we obtain the following master equation at steady-state :

$$0 = J(1-\phi)\delta_{k,1} + K((k+1)c_{k+1} - kc_k) - c_k \sum_{l=1}^{\infty} c_l + \frac{1}{2}\sum_{l=1}^{k-1} c_l c_{k-l} \tag{S11}$$

Where $J$ is the monomer injection rate per free (i.e occupied by a B component) site, $c_k$ is the average concentration of clusters of size $k$, and $\phi = \sum_{k=1}^{\infty} kc_k$ is the average surface fraction taken by the clusters. We can multiply this equation by $k$ and sum over all $k$ to find as in Section A.2 that at steady-state $\phi = J/(J+K)$. However, it is not possible to find an analytical expression for the concentration $c_k$. Nevertheless we can still find numerical solutions, an example of which is shown on Fig.1 of the main text. We see that the result is very similar to the one of Section A.2. The cluster size distribution is a power-law in $k^{-3/2}$ for small sizes and decreases exponentially beyond a cut-off size. In principle, a way to obtain such size using the distribution $c_k$ is to compute a ratio of this type :

$$S_n = \frac{\sum_{k=1}^{\infty} k^n c_k}{\sum_{k=1}^{\infty} k^{n-1} c_k} \tag{S12}$$

A different size $S_n$ is then obtained for each value of $n$. Let us consider the case $n = 2$. $S_2$ can be obtained from the knowledge of the first and second moment of the distribution $c_k$. For both recycling schemes, the first moment is given by $\phi = J/(J+K)$. The second moment $\sum_{k=1}^{\infty} k^2 c_k$ can be computed by multiplying the master equation (Eq.(S5) and Eq.(S11)) by $k^2$ and summing over $k$ . For the whole cluster recycling scheme we find :

$$S_2^c = \frac{\sum_{k=1}^{\infty} k^2 c_k}{\sum_{k=1}^{\infty} kc_k} = 1 + \frac{\phi}{K} \tag{S13}$$

We see that in the limit $\phi/K \gg 1$ (that is relevant throughout this paper), the size $S_2$ is proportional to the cut-off size $\kappa$ as defined in Eq.(S10). $S_2$ is therefore a good measure of the cut-off size of the distribution. In the monomer recycling case we find :

$$S_2^m = \frac{\sum_{k=1}^{\infty} k^2 c_k}{\sum_{k=1}^{\infty} kc_k} = 1 + \frac{\phi}{2K} \tag{S14}$$

We see that $S_2^m$ follows the same scaling in $\phi/K$ and differs only by a factor of two. Therefore in the infinite membrane approximation monomer recycling gives qualitatively the same results as whole cluster recycling.

### A.4 Steady-state size distribution: vesicular recycling

Here, we consider that recycling is performed by vesicles of a given size $n_v$ (in monomer unit). Clusters smaller than $n_v$ are recycled entirely, while larger clusters are fragmented by recycling, and recycled in patches of size $n_v$. The corresponding Master equation is given in the main text (Eqs.(1-2)):

$$\begin{aligned}\frac{dc_k}{dt} =& J(1-\phi)\delta_{k,1} - Kf\left(\frac{k}{n_v}\right)c_k + Kf\left(\frac{k+n_v}{n_v}\right)c_{k+n_v} \cdots \\ &\cdots \; - c_k\sum_{l=1}^{\infty} c_l + \frac{1}{2}\sum_{l=1}^{k-1} c_l c_{k-l}\end{aligned} \tag{S15}$$

where the function $f$ must represent the evolution of the recycling rate from a constant rate $K$ for $k < n_v$ (associated with the removal of whole clusters) to a rate $Kk/n_v$ for $k > n_v$ (associated with the removal of $n_v$ monomers from a cluster of size $k$). We take the following mathematical expression for $f$ :

$$f(x) = (1 + x^p)^{\frac{1}{p}} \tag{S16}$$

The power $p$ sets the "sharpness" of the transition between the two regimes. Here we have chosen $p = 5$. This more realistic scheme is clearly equivalent to monomer recycling if $n_v = 1$, and to whole cluster recycling if $n_v \to \infty$. Fig.1 of the main text shows how the corresponding size distribution crosses over smoothly from one extreme case to the other by varying $n_v$. This shows that a large class of recycling schemes may be understood on the basis of the results for the two extreme cases (which are qualitatively similar).

### A.5 Stochastic simulations : Validation of the infinite membrane prediction for large membrane sizes

Fig.S1 shows a comparison between the stochastic simulations of the cluster formation process with recycling in a situation for which the typical domain size $\kappa$ is well below the typical membrane size. The steady-state value of the concentration is obtained by averaging the results of a sufficiently large number of simulations. We see in this case that in the two situations studied (fixed area and fluctuating area) the infinite membrane result is satisfactory, thus validating this approach for systems without finite size effects.

## B Stochastic model for finite size systems

We now explore finite size effects for the whole cluster recycling scheme, both when the system size is constrained to a fixed value, or when it fluctuates with the incoming and outgoing fluxes. We then show in the fixed area case that the monomer recycling scheme gives qualitatively similar results.

## B.1 Whole cluster recycling

### B.1.1 Analytical model for finite size effects - fixed area

We artificially separate the population of clusters into a distribution of small size clusters and one large cluster of size $n(t)$ described by a probability distribution $p(n,t)$. For the small clusters we use the infinite membrane approach of Eq.(S5) and therefore we characterise their size distribution by the average concentrations $c'_k$. But we take into account the surface fraction $\phi_L$ occupied in average by the large cluster. Moreover, small clusters can disappear by getting recycled or by coalescing with the large cluster, therefore we replace $K$ by $K+1/N_s$. We get the following equation for the steady-state :

$$\begin{aligned} 0 = J(1-\phi_S-\phi_L)\delta_{k,1} - \left(K+\frac{1}{N_s}\right)c'_k \\ -c'_k\sum_{l=1}^{\infty}c'_l + \frac{1}{2}\sum_{l=1}^{k-1}c'_l c'_{k-l} \end{aligned} \tag{S17}$$

where $\phi_S = \sum_k k c'_k$ is the average surface fraction of the small clusters. Multiplying Eq.(S17) by $k$ and summing over $k$, we find that $\phi_S$ is given by :

$$\phi_S = \frac{J(1-\phi_L)}{J+K+N_s^{-1}} \tag{S18}$$

And $c'_k$ is simply given by Eq.(S9), in which $\phi$ is replaced by $\phi_S$ and $K$ is replaced by $K+1/N_s$. For the large cluster, we study the stochastic evolution of its size $n(t)$. Whenever the large cluster gets recycled, $n$ is set to zero and a new “large” cluster is recreated from scratch by coalescence events. We suppose that the surface fraction taken by the small clusters can be given at each time $t$ by Eq.(S18) with $\phi_L = n/N_s$. We know that the rate at which a cluster coalesce with the large one is $1/N_s$. Furthermore, if we make the approximation that the small clusters are all of size one, we find that $n$ verifies the following stochastic evolution :

$$\begin{cases} n \rightarrow n+1 & \text{At rate } \epsilon(N_s-n) \\ n \rightarrow 0 & \text{At rate } K \end{cases} \tag{S19}$$

where $\epsilon$ is given by $J/(1+N_s(J+K))$. Calling $p(n)$ the steady-state probability distribution of $n$ and using Eq.(S19), we can write the equations verified by $p(n)$ :

$$\begin{aligned} 0 &= -p(n)(K+\epsilon(N_s-n)) + p(n-1)\epsilon(N_s-n+1) \quad \forall n>0 \\ 0 &= -p(0)\epsilon N_s + (1-p(0))K \end{aligned} \tag{S20}$$

Solving these equations recursively gives the expression of $p(n)$ for all $n$ :

$$p(n) = \frac{KN_s!}{\epsilon(N_s-n)!}\prod_{k=0}^{n}\left(\frac{K}{\epsilon}+N_s-k\right)^{-1} \tag{S21}$$

From this we find the average surface fraction taken by the large cluster $\phi_L = \sum_n np(n) = (1 + K/\epsilon)^{-1}$, which together with Eq.(S18) enables to give self-consistent expressions for $\phi_S$ and $\phi_L$:

$$\phi_S = \frac{J}{J+K}\left(\frac{KN_s}{KN_s+1}\right), \quad \phi_L = \frac{J}{J+K}\left(\frac{1}{KN_s+1}\right) \tag{S22}$$

We note that the total surface fraction $\phi = J/(J+K)$ is unchanged in this mixed approach, compared to the infinite membrane approach. Moreover, when $N_s \to \infty$ we recover the infinite membrane solution as expected. We can find an anaytical approximation for Eq.(S21) using the fact that for $n \gg 1$, $\prod_{i=0}^{n}(x+i) \approx n! n^x/\Gamma(x)$ with $\Gamma(x) = \int_0^\infty t^{x-1}e^{-t}dt$. In the case $N_s \gg n \gg 1$, we obtain :

$$p(n) \approx \frac{K}{\epsilon N_s}\left(1 - \frac{n}{N_s}\right)^{\frac{K}{\epsilon}-1} = \frac{1}{N_s}\left(\frac{1-\phi}{\phi} + \frac{2N_s}{\kappa}\right)\left(1 - \frac{n}{N_s}\right)^{\frac{1-2\phi}{\phi}+\frac{2N_s}{\kappa}} \tag{S23}$$

The full size distribution for the fixed area model is given by:

$$c_{n,\text{fixed}} = c_n' + \frac{p(n)}{N_s} \tag{S24}$$

### B.1.2 Analytical model for finite size effects - fluctuating area

If the $B$ membrane component is not recycled, the membrane size is subjected to stochastic fluctuations and can be written $N_s = N_{s0} + \phi_S N_s + n$, with $N_{s0}$ the constant size occupied by the $B$ components, $\phi_S$ the surface fraction of small clusters and $n$ the size of the large cluster. Small clusters are as before treated in the infinite membrane approximation and we consider $\phi_S$ constant. Therefore we obtain the following average membrane size :

$$\langle N_s \rangle = \frac{N_{s0} + \langle n \rangle}{1 - \phi_S} \tag{S25}$$

For the small clusters the approach is very similar than in the previous case, except that in the expression of $\phi_S$ we replace $N_s$ by its average value given by Eq.(S25). From Eq.(S18), we find:

$$\phi_S = \frac{J(1-\phi_L)}{J + K + \frac{1-\phi_S}{N_{s0}+\langle n \rangle}} \tag{S26}$$

For the large cluster, as we consider that the surface fraction of small clusters is constant we have a simpler model for the evolution of $n$ than for the fixed area case. Indeed, the total mass contained in the small clusters is $\phi_S N_s$, and the coalescence rate with the large cluster is $1/N_s$. Therefore under this approximation the growth rate of the large cluster is constant and equal to $\phi_S$. So we have the following stochastic system :

$$\begin{cases} n \;\to\; n+1 & \text{At rate } \phi_S \\ n \;\to\; 0 & \text{At rate } K \end{cases} \tag{S27}$$

Again we can write the equations for the steady-state probability distribution $p(n)$ of $n$ :

$$\begin{aligned} 0 &= -p(n)(K+\phi_S) + p(n-1)\phi_S \quad \forall n > 0 \\ 0 &= -p(0)\phi_S + (1 - p(0))K \end{aligned} \tag{S28}$$

One can solve this equation recursively to obtain the expression of $p(n)$ :

$$p(n) = \frac{K}{\phi_S}\left(\frac{\phi_S}{\phi_S + K}\right)^{n+1} \tag{S29}$$

The value of $\langle n \rangle$ is therefore given by $\sum_n np(n) = \phi_S/K$. If we approximate the average surface fraction taken by the large cluster by $\langle n \rangle / \langle N_s \rangle$ we find another relation for $\phi_S$ and $\phi_L$ :

$$\phi_L = \frac{(1-\phi_S)\phi_S}{KN_{s0} + \phi_S} \tag{S30}$$

Eq.(S30) together with Eq.(S26) allows us to obtain the self-consistent expressions for $\phi_S$ and $\phi_L$ :

$$\begin{aligned} \phi_S &= \frac{JN_{s0}}{1 + N_{s0}(J+K)} \\ \phi_L &= \frac{J}{(J+K)(1+N_{s0}(J+K))} \end{aligned} \tag{S31}$$

We find again that the total surface fraction is $J/(J+K)$ and that we recover the infinite membrane result for $N_{s0} \to \infty$. The full size distribution for the fluctuating area model given by:

$$c_{n,\text{fluct}} = c_n' + \frac{p(n)(1-\phi_S)}{N_{s0} + n} \tag{S32}$$

### B.1.3 Comparison with the numerical solution

As shown in the text (Fig.2), the analytical hybrid model agrees very well with the numerical results of stochastic simulations in the limit of small concentration ($\phi \ll 1$). Fig.S2 shows the comparison between the analytical expressions of Eqs.(S23,S24) and Eqs.(S29,S32) and the results of stochastic simulations, in the fixed area and fluctuating area models. The fixed area model shows an interesting dependence on the protein surface fraction $\phi$. As can be gathered from the analytical expression for the probability distribution of the large cluster size (Eq.(S23)), the probability of finding a large cluster increases with the cluster size if $\phi > 1/(2(1 - N_s/\kappa))$ for the fixed area model, while it is a decreasing function of the cluster size at all $\phi$ in the fluctuating area model. This is indeed verified by our stochastic simulations. We hasten to stress that the Smoluchowski coagulation equation, Eq.(S5), is not physically valid for high surface fraction ($\phi \gtrsim 1/2$), since it treats the $A$ species as the minority phase. It is nevertheless interesting to see that the hybrid model we propose is able to reproduce the finite size effects as described by the Master equation also in the high $\phi$ regime, even though the master equation is not strictly valid in this limit. Furthermore, the effect of varying $\phi$ on the size distribution is already apparent at smaller surface fraction in the fixed area model.

## B.2 Analytical model for the monomer recycling scheme

We now investigate finite size effect in the monomer recycling scheme, restricting ourselves to the case of a fixed area for brevity (both fixed and fluctuating area give similar results for small surface fraction $\phi \ll 1$). The system dynamics are defined by the following rates:

$$\begin{cases} \text{Injection :} & B \xrightarrow{J} A_1 \\ \text{Growth :} & A_k + A_l \xrightarrow{1/N_s} A_{k+l} \\ \text{Removal :} & A_k \xrightarrow{Kk} A_{k-1} + B \end{cases} \tag{S33}$$

where $A_k$ denotes a cluster with $k$ monomer.

TO obtain an analytical expression of the probability distribution of cluster size, we proceed as in Section B.1.1 and artificially separate the system into two subsystems. We consider on one hand the clusters that are small enough to be treated in the infinite membrane approximation, and on the other hand one large cluster that is treated stochastically. The approach for the small clusters is exactly the same as in Section B.1.1. We use Eq.(S11) but we include the surface fraction of the large cluster and the fact that small clusters can disappear by coalescence with the large one. We get :

$$\begin{aligned} 0 = J(1-\phi_S-\phi_L)\delta_{k,1} - \left(Kk+\frac{1}{N_s}\right)c'_k + K(k+1)c'_{k+1} \\ -c'_k\sum_{l=1}^{\infty}c'_l + \frac{1}{2}\sum_{l=1}^{k-1}c'_lc'_{k-l} \end{aligned} \tag{S34}$$

Where $\phi_S = \sum_{k=1}^{\infty} kc'_k$ is the average surface fraction of the small clusters, $\phi_L$ is the average surface fraction of the large cluster and $1/N_s$ is the coalescence rate with the large cluster. We can find, using the now familiar summation over all $k$, that the value of $\phi_S$ is given exactly as in Section B.1.1 by Eq.(S18). Furthermore we can build a stochastic model for the evolution of the size $n$ of the large cluster following again the same reasoning as in Section B.1.1 :

$$\left\{ \begin{array}{lll} n & \to & n+1 \quad \text{At rate } \epsilon(N_s-n) \\ n & \to & n-1 \quad \text{At rate } Kn \end{array} \right. \tag{S35}$$

With $\epsilon = J/(1+N_s(J+K))$. Again we can write the equations verified by $p(n)$ :

$$\begin{array}{lll} 0 & = & -p(n)(Kn+\epsilon(N_s-n)) + p(n+1)K(n+1) + p(n-1)\epsilon(N_s-n+1) \quad \forall n>0 \\ 0 & = & -p(0)\epsilon N_s + p(1)K \end{array} \tag{S36}$$

It is possible to compute $p(n)$ recursively as a function of $p(0)$. The additional step is then to use the fact that $\sum_{n=0}^{\infty} p(n) = 1$ to determine explicitly $p(n)$. We obtain eventually :

$$p(n) = \frac{\epsilon^n}{\left(\frac{\epsilon}{K}+1\right)^{N_s} K^n} \cdot \frac{N_s!}{n!(N_s-n)!} \tag{S37}$$

From this expression we find $\phi_L = \sum_{n=1}^{N_s} np(n)/N_s$ and then $\phi_S$ as functions of $J$,$K$ and $N_s$. The result is the same as for cluster recycling and is given by Eq.(S22). Finally, the average cluster concentration is obtained as $c'_k + p(n)/N_s$. The resulting distributions are shown on Fig.S3.
The main difference with the whole cluster recycling scheme is that in the monomer recycling scheme, the large domain is stationary, grows in time by coalescence, and shrinks by recycling, but is never entirely removed from the system. This leads to a peaked distribution at large size. The fit shown on Fig.S3 seems very satisfactory for a small system but tends to be less accurate for a larger system. We identify two possible origins for this discrepancy. The analytical model makes two simplifying assumptions; that there is only one large domain, and that this domain grows by a unit size after each coalescence event. Both assumptions should be less valid for larger systems, and both could explain the deviations observed in Fig.S3 for large system size. As our main interest is to study finite size effects, and not to give a precise account of the crossover between the infinite membrane and small system size regimes, we did not attempt to improve the hybrid analytical model.

# C Computation of reaction efficiency

## C.1 Inside a subunit of the membrane

Here we develop the detailed derivation of our model of enzymatic reaction with confinement into clusters that cover a surface fraction $\phi$ of the membrane. We artificially divide the membrane into subunits, each of which encloses a cluster in its center that covers a fraction $\phi$ of its surface. We therefore assume cylindrical symmetry and we write diffusion/reaction equations both for the substraste $S$ (of concentration $c_S$ and intermediate $I$ (of concentration $c_I$) inside a subunit of radius $R_c$ containing a circular cluster of radius $R_d = R_c\sqrt{\phi}$ :

$$\begin{aligned} \frac{dc_S}{dt} &= K_0 - \beta c_S - c_S\alpha\Theta(R_d - r) + D_m\Delta c_S \\ \frac{dc_I}{dt} &= -\beta c_I + c_S\alpha\Theta(R_d - r) - c_I\alpha\Theta(R_d - r) + D_m\Delta c_I \end{aligned} \tag{S38}$$

The substrate $S$ is injected uniformly on the membrane at a rate $K_0$, diffuses freely with a diffusion constant $D_m$ (identical in and outside the domains) and is turned in the intermediate $I$ by enzymes $E_1$ present inside the membrane clusters. The reaction kinetics is assumed to be a linearised Michaelis-Menten kinetics, thus proportional to the substrate concentration $c_S$. The value of the proportionality constant is $\alpha$. The situation is identical for the intermediate $I$, which diffuses freely and is transformed into the product $P$ upon encounter the enzymes $E_2$ (which is also only present inside the cluster) using the same kinetic parameters. Furthermore we assume that both $S$ and $I$ can be degraded everywhere on the membrane at a rate $\beta$. Now we define $\tilde{c} = c\beta/K_0$ as the dimensionless concentration and $\tilde{r} = r\sqrt{\beta/D_m}$ as the dimensionless length. Furthermore we define $\mu = \alpha/\beta$.

For $0 < \tilde{r} < \tilde{R}_d$ we then have :

$$\begin{aligned} \frac{d\tilde{c}_S}{dt} &= 1 - \tilde{c}_S(1+\mu) + \Delta\tilde{c}_S \\ \frac{d\tilde{c}_I}{dt} &= -\tilde{c}_I(1+\mu) + \tilde{c}_S\mu + \Delta\tilde{c}_I \end{aligned} \tag{S39}$$

And for $\tilde{R}_d < \tilde{r} < \tilde{R}_c$ :

$$\begin{aligned} \frac{d\tilde{c}_S}{dt} &= 1 - \tilde{c}_S + \Delta\tilde{c}_S \\ \frac{d\tilde{c}_I}{dt} &= -\tilde{c}_I + \Delta\tilde{c}_I \end{aligned} \tag{S40}$$

The boundary conditions require equality of concentration and fluxes at the domain boundaries, and a vanishing flux at the unit boundaries :

$$\left.\frac{d\tilde{c}_{I/S}}{d\tilde{r}}\right|_0 = \left.\frac{d\tilde{c}_{I/S}}{d\tilde{r}}\right|_{\tilde{R}_c} = 0, \qquad \left.\frac{d\tilde{c}_{I/S}}{d\tilde{r}}\right|_{\tilde{R}_d^-} = \left.\frac{d\tilde{c}_{I/S}}{d\tilde{r}}\right|_{\tilde{R}_d^+}, \quad \tilde{c}_{I/S}(R_d^-) = \tilde{c}_{I/S}(R_d^+) \tag{S41}$$

## C.2 Efficiency definition and computation in the homogeneous case

The efficiency $\eta$ of the reaction is measured by comparing the production flux of P to the injection flux of S at steady-state. For a subunit, this translates to :

$$\eta = \frac{\int_{r<R_d} d\vec{r} c_I(\vec{r})\alpha}{K_0 \pi R_c^2} = \frac{2\mu\phi}{\tilde{R}_d^2} \int_0^{\tilde{R}_d} \tilde{r}\tilde{c}_I(\tilde{r}) d\tilde{r} \tag{S42}$$

We see that $\eta$ does not depend on the source term $K_0$ but only on $\tilde{R}_d$, $\mu$ and $\phi$. If enzymes are homogeneously (which corresponds to very small domains, $R_d \to 0$, reactions with enzymes are permitted everywhere in the system but with a smaller rate $\phi\alpha$ due to dilution. The steady state values of the concentrations are $c_S = 1/(1+\phi\mu)$ and $c_I = \phi c_S/(1+\phi\mu)$. The value of the efficiency in the homogeneous case is then :

$$\eta_0 = \left(\frac{\phi\mu}{1+\phi\mu}\right)^2 \tag{S43}$$

## C.3 Expression of $\eta$

In the general case with clusters of radius $R_d$, by integrating Eq.(S39) and Eq.(S40) one finds the expression of $\eta$ as a function of two derivatives :

$$\eta = \frac{\phi\mu}{1+\mu}\left(\frac{2}{\tilde{R}_d} \cdot \left.\frac{d\tilde{c}_I}{d\tilde{r}}\right|_{\tilde{R}_d} + \frac{\mu}{1+\mu}\left(1 + \frac{2}{\tilde{R}_d} \cdot \left.\frac{d\tilde{c}_S}{d\tilde{r}}\right|_{\tilde{R}_d}\right)\right) \tag{S44}$$

Eq.(S39) and Eq.(S40) allow to compute analytically the expression of $\frac{d\tilde{c}_S}{d\tilde{r}}$ at steady-state using modified Bessel functions (the tildes are omitted henceforth for simplicity) :

$$\left.\frac{dc_S}{dr}\right|_{R_d} = \mathfrak{C}\sqrt{1+\mu} I_1\left(\sqrt{1+\mu}R_d\right) \tag{S45}$$

The integration constant $\mathfrak{C}$ is defined by the following relation :

$$\frac{\mathfrak{C}\sqrt{1+\mu} I_1\left(\sqrt{1+\mu}R_d\right)}{\mathfrak{C} I_0\left(\sqrt{1+\mu}R_d\right) - \frac{\mu}{1+\mu}} = \mathfrak{F}(R_d, R_c) \tag{S46}$$

In which $\mathfrak{F}$ is given by :

$$\mathfrak{F}(R_d, R_c) = \frac{I_1(R_d) - \frac{I_1(R_c)}{K_1(R_c)} K_1(R_d)}{I_0(R_d) + \frac{I_1(R_c)}{K_1(R_c)} K_0(R_d)} \tag{S47}$$

Now in order to obtain $\frac{dc_I}{dr}$ we need to solve Eq.(S39) and Eq.(S40) for $c_I$ with a spatially inhomogeneous source term given by $c_S(r)$. The solution is given by :

$$c_I(r) = \frac{\mu}{(1+\mu)^2} + \mathfrak{B} \cdot I_0(\sqrt{1+\mu}r) - \mathfrak{C} \cdot \frac{\mu}{2\sqrt{1+\mu}} r I_1(\sqrt{1+\mu}r) \tag{S48}$$

Where the constant $\mathfrak{C}$ is given by Eq.(S46) and the constant $\mathfrak{B}$ is given by the fraction $\mathfrak{B}_1/\mathfrak{B}_2$ with:

$$\begin{aligned}\mathfrak{B}_1 &= \mathfrak{F}\left(\frac{\mu}{(1+\mu)^2} - \mathfrak{C}\frac{R_d\mu}{2\sqrt{1+\mu}}I_1(\sqrt{1+\mu}R_d)\right) + \mathfrak{C}\frac{R_d\mu}{2}I_0(\sqrt{1+\mu}R_d) \\ \mathfrak{B}_2 &= \sqrt{1+\mu}I_1(\sqrt{1+\mu}R_d) - \mathfrak{F}I_0(\sqrt{1+\mu}R_d) \end{aligned} \tag{S49}$$

From these equations we find the exact analytical expression of $\eta$, which depends only on $\mu$, $R_d$ (still dimensionless) and $\phi$ :

$$\eta = \frac{\phi\mu}{1+\mu}\left(\frac{\mu}{1+\mu} - \mathfrak{C}\mu I_0(\sqrt{1+\mu}R_d) + \frac{2\sqrt{1+\mu}I_1(\sqrt{1+\mu}R_d)}{R_d}\left(\mathfrak{B} + \mathfrak{C}\frac{\mu}{1+\mu}\right)\right) \tag{S50}$$

### C.4 Qualitative and quantitative features of $\eta(R_d)$

As is apparent from Fig.4 in the main text, the domain size dependence of the enzymatic efficiency is non-trivial and not necessarily monotonic. Here we derive to conditions under which efficiency is optimised for small, large, or intermediate-size domains. To do this, we need to obtain the behaviour of $\eta(R_d)$ for $R_d \ll 1$ and $R_d \gg 1$. The following expansions can be obtained from Eq.(S50):

$$\text{For} \quad R_d \ll 1, \quad \eta \approx \frac{\phi^2\mu^2}{(1+\phi\mu)^2} + R_d^2\frac{\phi\mu^2}{4(1+\phi\mu)^3}(\phi\mu - 1)(\ln\phi + 1 - \phi) \tag{S51}$$

$$\text{For} \quad R_d \gg 1, \quad \eta \approx \frac{\phi\mu^2}{(1+\mu)^2} + \frac{\phi\mu^2(2\mu^2 + \mu + 3\sqrt{1+\mu}\mu - 4\sqrt{1+\mu} - 4)}{R_d(\sqrt{1+\mu}+1)^3(1+\mu)^{\frac{5}{2}}} \tag{S52}$$

From this we can prove the following statements :

1. $\phi < min(\frac{1}{\mu^2}, 1) \Leftrightarrow \eta(0) < \eta(\infty)$
   **Proof :** We see using Eq.(S52) that $\eta(\infty) = \frac{\phi\mu^2}{(1+\mu)^2}$ which leads to
   $$\eta(0) < \eta(\infty) \Leftrightarrow 1 - \phi(1+\mu^2) + \phi^2\mu^2 > 0 \tag{S53}$$
   Solving the polynomial equation leads directly to the result.
2. $\phi < \frac{1}{\mu} \Leftrightarrow \eta(R_d \ll 1)$ is increasing.
   **Proof :** The dominant non-constant term in Eq.(S51) is in $R_d^2$, therefore $\eta(R_d \ll 1)$ is increasing if the multiplicative factor in front of $R_d^2$ is positive. This constraint is written as :
   $$(\phi\mu - 1)(\ln\phi + 1 - \phi) > 0 \tag{S54}$$
   $\forall\phi \in [0, 1]$ we have $\ln\phi + 1 - \phi < 0$ therefore $\eta$ is increasing for $R_d \ll 1$ if $\phi < \frac{1}{\mu}$.
3. $\mu > \frac{5}{4} \Leftrightarrow \eta(R_d \gg 1)$ is decreasing.
   **Proof :** Here, $\eta(R_d \gg 1)$ is decreasing if the multiplicative factor in front of $1/R_d$ in Eq.(S52) is positive :
   $$2\mu^2 + \mu + 3\mu\sqrt{1+\mu} - 4\sqrt{1+\mu} - 4 > 0 \tag{S55}$$

The expression in Eq.(S55) can be differentiated and is found to be and increasing function of $\mu$. Therefore $\mu$ needs to be larger than a critical value for which the expression is equal to zero. Isolating the $\sqrt{1+\mu}$ factor and elevating to power two leads to a polynomial equation which after simplification is reduced to the trivial equation $4\mu - 5 = 0$. Therefore $\eta$ is decreasing for large values of $R_d$ if $\mu > \frac{5}{4}$.

In short, there is an optimal cluster size if and only if $\mu > \frac{5}{4}$ and $\phi < \frac{1}{\mu}$. The efficiency gain at the maximum is an increasing function of $\mu$.

### C.5 Computation of the global efficiency $\eta^\star$

So far, the efficiency was computed for a single unit with circular symmetry. The full efficiency of a membrane where domain components constantly recycle to the cytoplasm, and where the spatial distribution of domain in the membrane is arbitrary, is very complicated to calculate. Here, we take a simple approach and assume that the dynamics of cluster reorganisation is very slow compared to the dynamics of reaction and diffusion of S, I and P. Under this assumption we can compute the global efficiency for each configuration of clusters, assuming steady-state concentration profiles. It means that at each time $t$, provided that we know the size distribution of clusters, we can compute an instantaneous value of the efficiency that we call $\eta^\star_{ins}(t)$. Since membrane clusters are continuously inserted, removed and can coalesce with each other., the cluster size distribution is a fluctuating quantity, and so is $\eta^\star_{ins}(t)$. Therefore, we define the global efficiency $\eta^\star$ of the system as the time average of $\eta^\star_{ins}(t)$. To simplify the computation we restrict ourselves to the case of a fixed membrane area of size $N_s$, which is populated at time $t$ by $n_i(t)$ clusters of size $i$ (i.e made of $i$ elementary units) with an overall surface fraction $\phi(t)$. In the context of enzymatic reactions, we can consider that an elementary unit is made of one enzyme of each type surrounded by a small number of lipids forming a different phase than the bulk lipids. Now, in order to compute the efficiency of the system we will divide it into subunits as described in the previous sections, a fraction $\phi(t)$ of the surface of each being taken by one cluster. We can express the total efficiency $\eta^\star_{ins}(t)$ by summing the contributions $R_i^{I\to P}$ of each subunit $i$ to the total production rate of P :

$$\eta^\star_{ins}(t) = \frac{1}{K_0 S} \sum_{i \in subunits} R_i^{I\to P} \tag{S56}$$

$S$ being the total surface of the membrane and $K_0$ the injection rate of S. We make now the approximation that we can compute $R_i^{I\to P}$ using the computation that we made previously of $\eta$. It means that we suppose that all the subunits can be treated as if they were all cylindrically symmetrical with no flux conditions at the boundary. The approximation is reasonnable in the small density limit ($\phi \ll 1$) where clusters are far apart. Under these assumptions, we define $S_i$ the surface of the subunit $i$ and we have :

$$\frac{R_i^{I\to P}}{K_0 S_i} = \eta\left(\sqrt{\frac{\phi S_i}{\pi}}, \phi\right) \tag{S57}$$

Which leads to :

$$\eta^\star_{ins}(t) = \sum_{i \in subunits} \frac{S_i}{S}\eta\left(\sqrt{\frac{\phi S_i}{\pi}}, \phi\right) \tag{S58}$$

This expression can be rewritten to sum over the cluster sizes (1,2,...,$N_s$) :

$$\eta^{\star}_{ins}(t) = \sum_{k=1}^{N_s} \frac{k n_k(t)}{N_s \phi(t)} \eta \left( \sqrt{\frac{ks}{\pi}}, \phi(t) \right) \tag{S59}$$

The global efficiency $\eta$ of the system is the time average of Eq.(S59). We see that the result depends on the statistical properties of the size distribution $n_k$. As we would like to be able to compute $\eta$ only as a function of the average size distribution $c_k = \langle n_k \rangle / N_s$, we use the mean-field average of Eq.(S59) which gives the following formula :

$$\eta^{\star} = \sum_{k=1}^{N_s} \frac{k c_k}{\langle \phi \rangle} \eta \left( \sqrt{\frac{ks}{\pi}}, \langle \phi \rangle \right) \tag{S60}$$

with $\langle \phi \rangle = \sum_k k c_k$. This approximation thus gives an estimation of the efficiency of the system using only the knowledge of the average cluster size distribution.
