# Supplementary figures and images for: Sensing Size through Clustering in Non-Equilibrium Membranes and the Control of Membrane-Bound Enzymatic Reactions
Source: PLoS One. 2015 Dec 14;10(12):e0143470. doi: 10.1371/journal.pone.0143470 (PMC4687633; doi:10.1371/journal.pone.0143470)

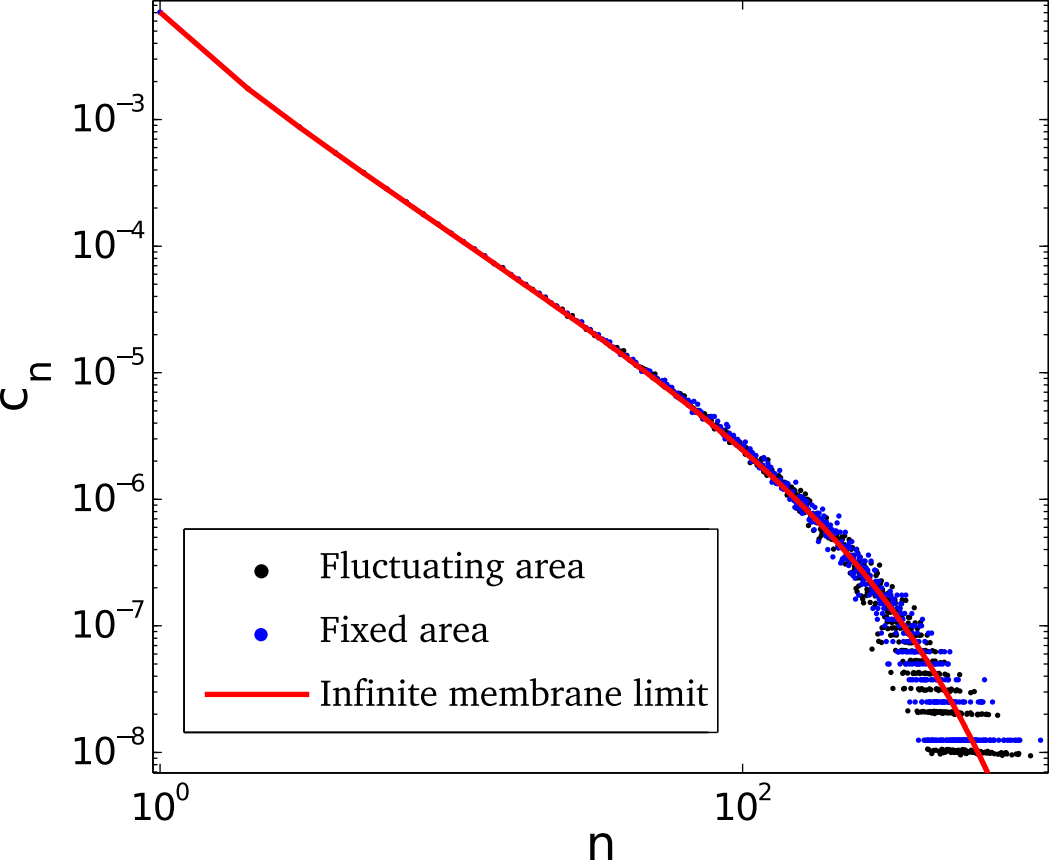

Supplement: S1 Fig — Domain size distributions at steady-state (t = 5.104, averaged over 20000 realisations) for the fixed and fluctuating area models with ϕ = 0.1 and κ = 200. The membrane size is either fixed at N s = 4000 or initially set at N s0 = 4000. The red line is the infinite membrane result. (PDF) [file pone.0143470.s002.pdf]

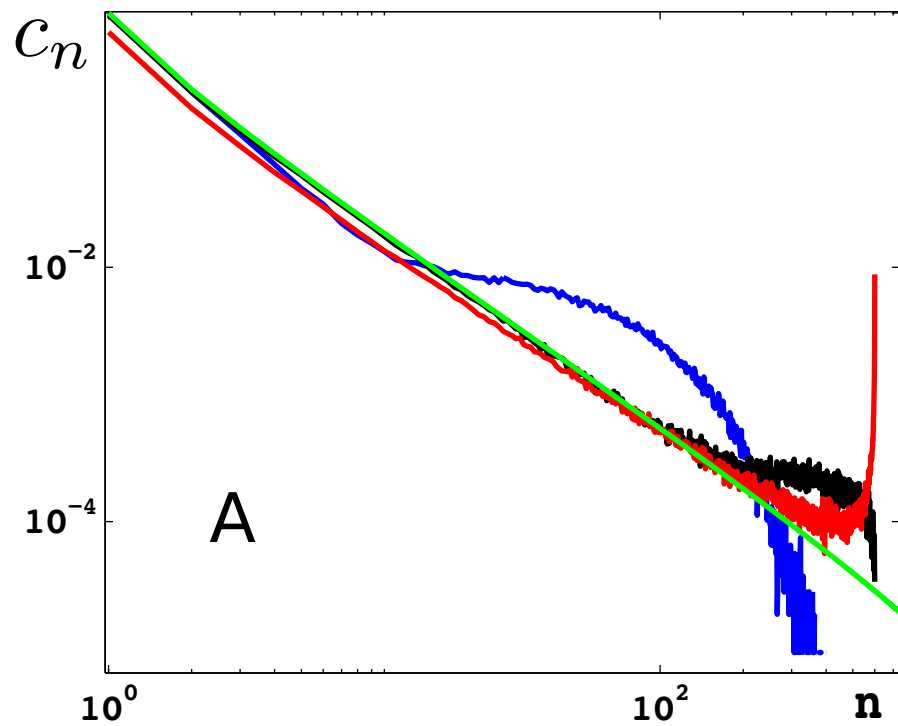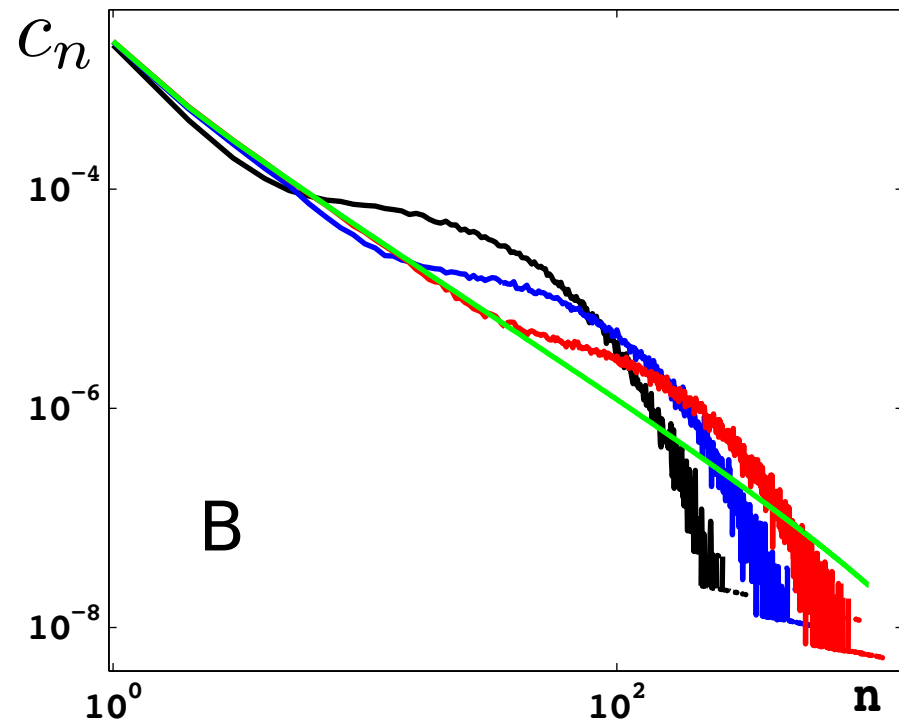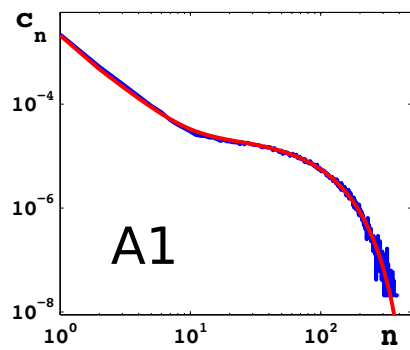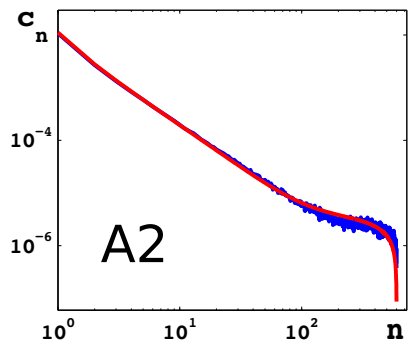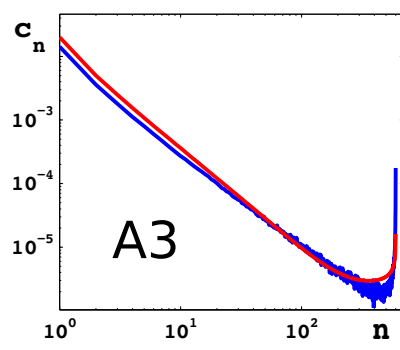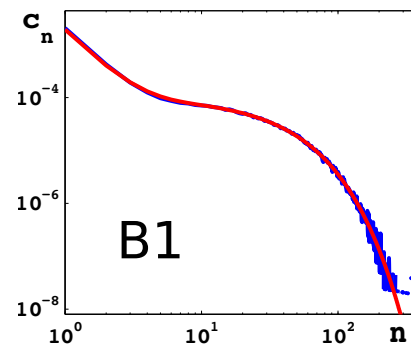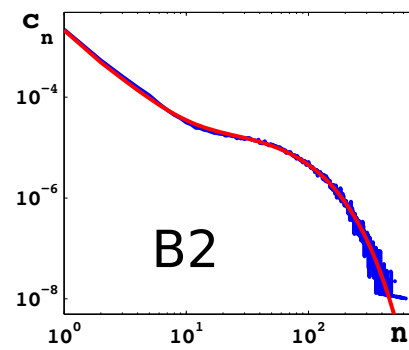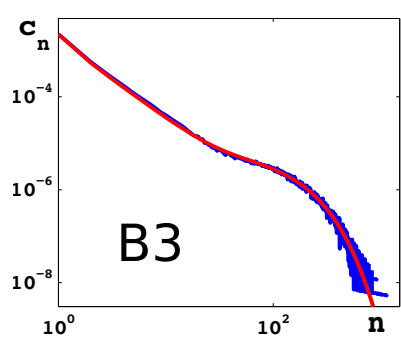

Supplement: S2 Fig — Steady-state cluster size distributions obtained by simulations compared to the infinite membrane result (in green). (A) Results for the fixed area model (N s = 600, κ = 2000), for the steady-state surface fraction ϕ equal to 0.1 (blue), 0.5 (black) and 0.9 (red). Panels A1–A3: Comparison of the simulation results (blue) with the analytical predictions of Eqs (23, 24) of S1 File. (red) for the three surface fractions. (B) Results for the fluctuating area model (ϕ = 0.1, κ = 2000) with the initial membrane size N s0 equal to 300 (black), 600 (blue) and 1200 (red). Results (not shown) at other surface fractions show no qualitative difference. Panels B1–B3: Comparison of the simulation results (blue) with the analytical predictions of Eqs (29, 32) of S1 File (red) for the three initial sizes. (PDF) [file pone.0143470.s003.pdf]

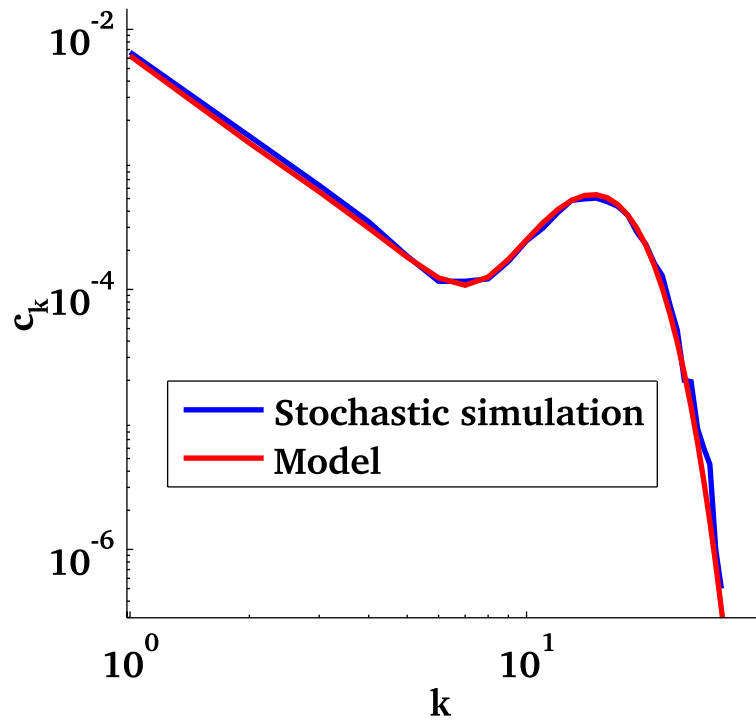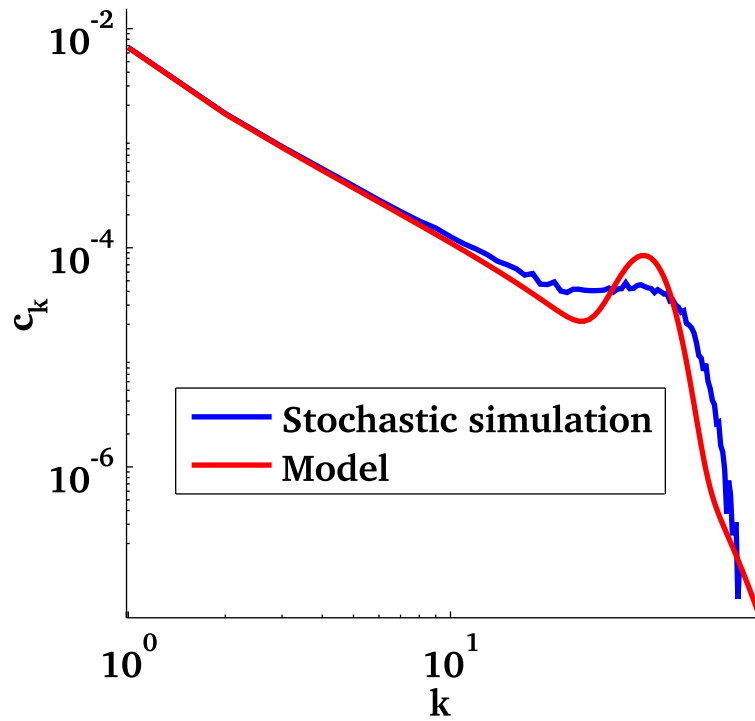

Supplement: S3 Fig — Steady-state size distributions using the monomer recycling mechanism for a fixed area. The injection rate is J = 10−4 and the recycling rate is K = 10−3. On the left panel the system size is set to N s = 200 and on the right panel the system size is set to N s = 800. The blue curves are stochastic simulation results extracted from Fig 2b of the main text and the red curves are the results of the analytical study. (PDF) [file pone.0143470.s004.pdf]
